# Supplementary material for: Label-free nanofluidic scattering microscopy of size and mass of single diffusing molecules and nanoparticles
Source: Nat Methods. 2022 May 30;19(6):751–8. doi: 10.1038/s41592-022-01491-6 (PMC9184284; doi:10.1038/s41592-022-01491-6)
Supplement: Supplementary file 1 — Supplementary Sections 1–12. [file 41592_2022_1491_MOESM1_ESM.pdf]

nature portfolio

https://doi.org/10.1038/s41592-022-01491-6

Supplementary information

Label-free nanofluidic scattering microscopy of size and mass of single diffusing molecules and nanoparticles

In the format provided by the authors and unedited

Supplementary Information

Label-Free Nanofluidic Scattering Microscopy of Size and Mass of Single Diffusing Molecules and Nanoparticles

Barbora Špačková[1]*, Henrik K. Moberg[1], Joachim Fritzsche[1], Johan Tenghamn[1], Gustaf Sjösten[2], Hana Šípová-Jungová[1], David Albinsson[1], Quentin Lubart[3], Daniel van Leeuwen[3], Fredrik Westerlund[3], Daniel Midtvedt[2], Elin K. Esbjörner[3], Mikael Käll[1], Giovanni Volpe[2], Christoph Langhammer[1]**

[1]Department of Physics, Chalmers University of Technology; 412 96 Göteborg, Sweden

[2]Department of Physics, University of Gothenburg; 412 96 Göteborg, Sweden

[3]Department of Biology and Biological Engineering, Chalmers University of Technology; 412 96 Göteborg, Sweden

*Corresponding author. Email: spackova@chalmers.se

**Corresponding author. Email: clangham@chalmers.se

1. Light scattering of a biomolecule inside a nanochannel

1.1 Scattering intensity

Elastic scattering of light is a fundamental process in light-matter interactions. For three-dimensional nano-objects, such as biomolecules or nanoparticles, whose size is much smaller than the wavelength of the incident light (λ), this process is known as Rayleigh scattering. From here forward we explicitly refer to biomolecules only. However, we highlight that the solution we have developed is generic and also applies to other nano-objects equally well. The total scattering intensity (power), that is collected by the optical system,

$$P_m = cI_0\sigma_m,$$

Equation 1

is determined by the incident intensity (I_0), the collection efficiency (c), and the scattering cross-section of a biomolecule (σ_m), defined as[1]

$$\sigma_m = \frac{k^4}{6\pi}|\alpha_m|^2,$$

Equation 2

where $k = 2\pi n/\lambda$ is the wavenumber of light in the medium with refractive index n, and α_m is the polarizability of the biomolecule,

$$\alpha_m = V_m \frac{n_m^2 - n^2}{n^2 + \nu(n_m^2 - n^2)}$$

Equation 3

where n_m is the refractive index of the biomolecule, V is its volume, and ν is the depolarization factor, depending on its shape (for a sphere $\upsilon = 1/3$, for an ellipsoid υ ranges between 0 and $1/2$ depending on the ratio of its semiaxis).

A nanochannel, which is an object of finite (subwavelength) size in only two dimensions, can scatter light as well. It can be approximated as a cylinder with cross sectional area (A) and length that is much larger than $\sqrt{A}$. The refractive index of the inside, n_i, is lower than the refractive index of the outside, n_o. The scattering intensity is constant along the length of such a nanochannel, and the total scattering intensity can be written as

$$P_c = cI_0L\sigma_c,$$

Equation 4

where L is the length of the nanochannel from which the scattered light is collected and σ_c is the scattering cross section of the nanochannel. For a nanochannel having circular cross-section, σ_c can then be determined by the analytical expression presented by Mie[1]. For a nanochannel with $\sqrt{A} \ll \lambda$ (Rayleigh limit), and light incidence angle perpendicular to its long axis, the solution can be simplified to[1]

$$\sigma_c = \frac{k_o^3}{4}|\alpha_c|^2$$

Equation 5

where k_o is the wavenumber in the medium outside the nanochannel, $k_o = 2\pi n_o/\lambda$, and

$$\alpha_c = A\frac{n_i^2 - n_o^2}{n_o^2 + \nu(n_i^2 - n_o^2)}$$

Equation 6

is the one-dimensional polarizability of the nanochannel, where ν is the depolarization factor that depends on the shape of the nanochannel (for a cylinder with aspect ratio equal 1, $\upsilon = 0$ for TM polarization, $\upsilon = 1/2$ for TE polarization).

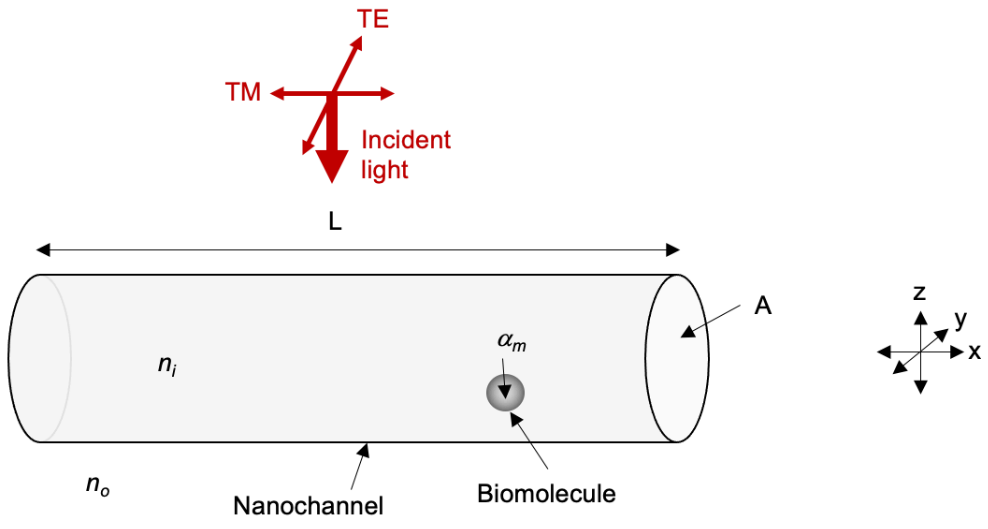

Fig. S1. Schematic of the assumed scenario: *a cylindrical nanochannel with a single biomolecule inside.*

When a biomolecule is present inside such a nanochannel (**Fig. S1**), the combined scattering by the nanochannel and the biomolecule becomes a complex canonical problem of scattering by a sphere embedded inside a cylinder. The analytical solution to this problem was recently presented, based on the expansion of cylindrical waves into spherical ones and vice versa[2]. However, it leads to a series of rather complex expressions, from which it is difficult to draw a more generalized conclusion. Therefore, we instead employ the Clausius-Mossotti relation that describes the relation between the microscopic quantity — polarizability — and the macroscopic quantity — dielectric function. It states that the contribution of biomolecules enclosed in a cavity can be described in terms of a bulk optical property of the material inside the cavity — the effective permittivity[3]

$$\varepsilon^* \approx n_i^2\left(1 + \frac{\alpha_m n_V}{1 - \upsilon\alpha_m n_V}\right),$$

Equation 7

where n_V is the volume density of the biomolecules, $n_V = N/V$, where N is the number of biomolecules in the volume V.

Using the expression for the polarizability of a cylinder (Equation 6), where the refractive index of the inside is assumed to be $n_i \approx \sqrt{\varepsilon^*}$ (Equation 7), the total polarizability of a nanochannel comprising a single biomolecule can then be written as

$$\alpha_t^{TM} = \mathrm{A}\left[\frac{n_i^2}{n_o^2} - 1 + \frac{n_i^2}{n_o^2} n_V \alpha_m\right]$$

Equation 8

for a cylinder with circular cross-sectional and TM polarization and

$$\alpha_t^{TE} = \mathrm{A}\left[\frac{2\frac{n_i^2 - n_o^2}{n_i^2 + n_o^2} + n_V\alpha_m}{1 + \frac{n_V\alpha_m}{2}\frac{n_i^2 - n_o^2}{n_i^2 + n_o^2}}\right]$$

Equation 9

for a cylinder with circular cross-sectional and TE polarization.

For biomolecules with low polarizability that are sparsely distributed inside the nanochannel, $n_V\alpha_m \ll 1$, and a small difference between the refractive indexes of the outside and inside of the nanochannel, $(n_i^2 - n_o^2) < (n_i^2 + n_o^2)$, the second term in the denominator in Equation 9 can be neglected and polarizability of the nanochannel containing the biomolecule (Equation 8 and Equation 9) can be written as

$$\alpha_t = \alpha_c + m\,\alpha_m/L,$$

Equation 10

where $m = 1$ for TE polarization and $m = n_i^2/n_o^2$ for TM polarization. The scattering cross section of the nanochannel containing the biomolecule can then be written as

$$\sigma_t = \frac{k_o^3}{4}|\alpha_t|^2.$$

Equation 11

In order to confirm the conclusions of the analytical theory presented above, we have carried out finite-difference time-domain (FDTD) simulations. They were performed using the Lumerical FDTD Solutions software package. The simulated structure — a cylindrical nanochannel with radius r_c — was placed into a 3D simulation cell of the size $L \times 0.5 \times 0.5$ μm^3. The refractive indexes of the inside and outside of the nanochannel correspond to water (n_i = 1.33 RIU) and SiO_2 (n_o = 1.46 RIU), respectively. Biomolecules were simulated as dielectric spheres with refractive index n_m = 1.43 and radius r_m. Periodic boundary conditions were set in the x direction and perfect matching layer absorbing boundary conditions were set in the y and z directions. The mesh step was 1 nm. Light was introduced as a linearly polarized plane wave via a total-field/scattered-field source with normal incidence. If not stated otherwise, the wavelength of the incident light was λ = 500 nm. The scattered light was collected from 4 near-field monitors surrounding the nanochannel in all directions.

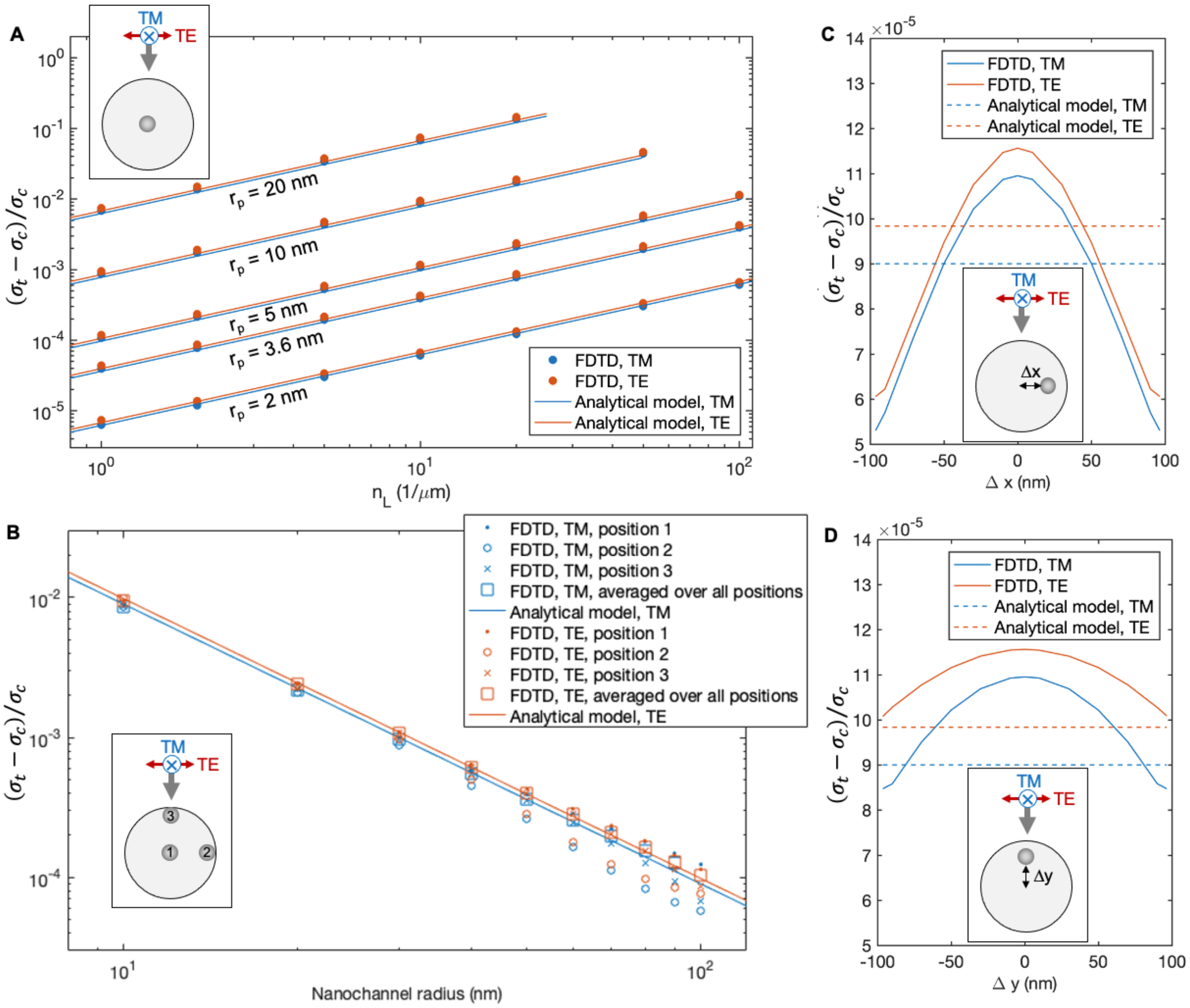

Fig. S2. Simulated relative difference between the scattering cross section of a nanochannel containing a biomolecule, and an empty nanochannel — $(\sigma_t - \sigma_c)/\sigma_c$. *A calculation using an analytical model (Equation 10 and Equation 11) is compared with FDTD simulations. The insets in the figures show schematics of the different calculated/simulated scenarios in a cross-sectional view.* ***(A)*** *Dependency of $(\sigma_t - \sigma_c)/\sigma_c$ on the linear density of biomolecules (n_L= N/L) for a nanochannel with radius r_c = 50 nm for TM and TE polarization and for a range of biomolecules defined by their radius (r_p). The biomolecules were assumed to be in the centre of the nanochannel.* ***(B)*** *Dependency of $(\sigma_t - \sigma_c)/\sigma_c$ on the radius of a nanochannel (r_c) for TM and TE polarization and a biomolecule with r_p = 3.6 nm and n_L = 10 μm^{-1} inside for different geometric scenarios with respect to the position of the molecule inside the channel (see inset): (i) in the centre, (ii) at the top, (iii) at the side and (iv) averaged over all possible positions.* ***(C, D)*** *Dependency of $(\sigma_t - \sigma_c)/\sigma_c$ on the (C) horizontal and (D) vertical position of a biomolecule with r_p = 3.6 nm and n_L = 0.1 μm^{-1} inside a nanochannel with r_c = 100 nm for TM and TE polarization.*

Fig. S2 shows the results of the FDTD simulations compared with the results of the analytical model (Equation 10 and Equation 11), presented as dependency of the relative difference of the scattering cross section of a nanochannel containing a biomolecule and an empty nanochannel, $(\boldsymbol{\sigma_t} - \boldsymbol{\sigma_c})/\boldsymbol{\sigma_c}$, on a variety of properties of the nanochannel and the biomolecule: polarizability (radius) and linear density of biomolecules ($\boldsymbol{n_L} = \boldsymbol{N/L}$) (**Fig. S2A**), cross-sectional dimension of the nanochannel (**Fig. S2B**), and axial position of the biomolecule inside the nanochannel (**Fig. S2B, C, D**). The overall trend captured by both approaches is in excellent quantitative agreement (**Fig. S2A**). However, the FDTD results show an additional dependency on the axial position of the biomolecule inside the nanochannel that is neglected by the analytical model and that originates from the phase difference between the light scattered by the nanochannel and the biomolecule (**Fig. S2B, C, D**). As a result, when a biomolecule freely diffuses inside the nanochannel, the scattering cross section randomly fluctuates over time, with the frequency depending on the diffusivity of the molecule (*D*). However, the mean value over all possible locations is very close to the analytical model (**Fig. S2B**). This means that if the mean travel distance, $\sqrt{2D\Delta t}$, within one integration time step (Δt) is much larger than the dimensions of the nanochannel cross section, the phase differences are effectively averaged, and the obtained scattering cross section value corresponds very well to the analytical model.

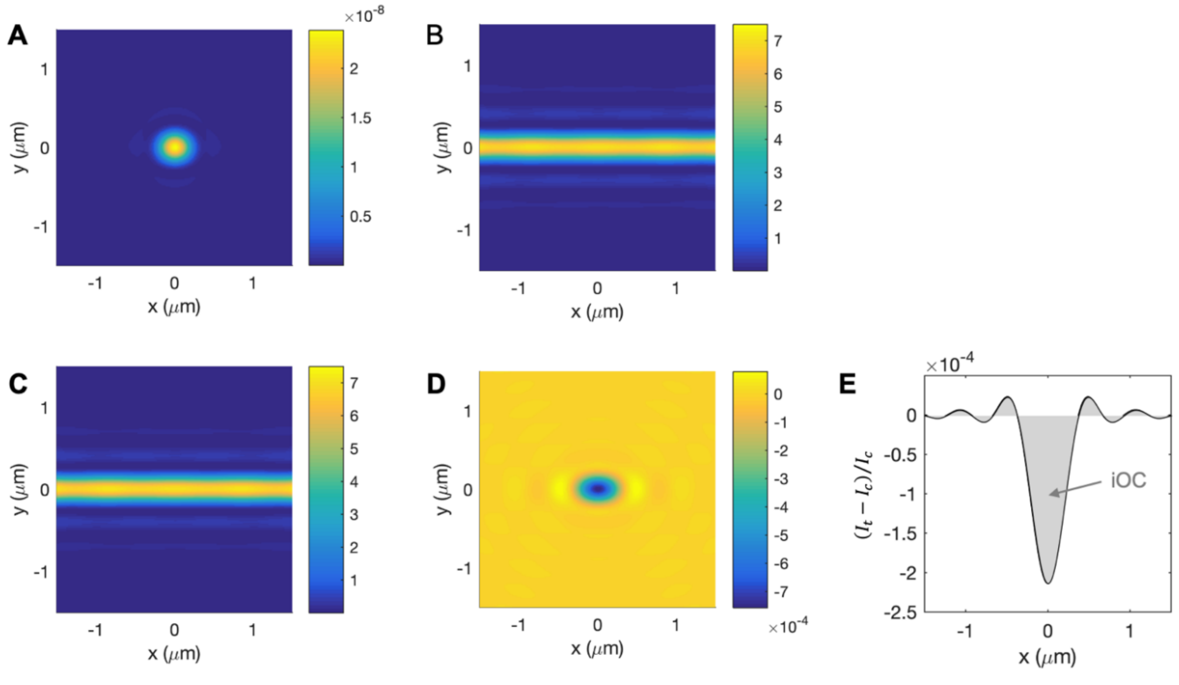

Fig. S3. Simulated dark-field image *of* ***(A)*** *a biomolecule,* ***(B)*** *a nanochannel,* ***(C)*** *the biomolecule inside the nanochannel.* ***(D)*** *The differential dark-field image obtained by subtracting an image of the empty nanochannel from the image of the nanochannel with the biomolecule inside.* ***(E)*** *Intensity profile of the differential dark-field image normalized by the intensity profile of the dark-field image of an empty nanochannel. Its integrated value corresponds to the integrated optical contrast (iOC) that is directly connected to polarizability (Equation 17) and can be used to estimate the molecular weight of a biomolecule (Equation 30). The nanochannel was simulated as a cylinder with* r_c *= 50 nm,* n_i *= 1.33 RIU, and* n_o *= 1.46 RIU. The biomolecule was simulated as a spherical particle with* r_p *= 3.6 nm and* n_m *= 1.46 RIU. The polarization was set to TM and NA = 1.*

The total scattering intensity, $P_t = cI_0L\sigma_t$, that is collected from a region of a nanochannel of the length $L_k = 3\pi/(2k_o m)$ containing a biomolecule can be written as

$$P_t = P_c + P_m + 2\text{sign}(n_i - n_o)\sqrt{P_c P_m},$$

Equation 12

using Equation 10 and Equation 11, where P_c and P_m correspond to the total scattering intensity of the nanochannel (Equation 1) and the biomolecule alone (Equation 4), respectively. Here, it has to be noted that the scattering intensity collected from a typical biomolecule (**Fig. S3A**) is several orders of magnitude lower than the scattering intensity collected from a typical nanochannel (**Fig. S3B**), $P_c \gg P_m$, because the volume contributing to the detected intensity is much larger than the molecular volume, $L_k A \gg V_m$. Therefore, the scattering signal stemming from the biomolecule that resides inside the nanochannel is not directly distinguishable (**Fig. S3C**), $P_t \approx P_c$. However, the scattering intensity integrated from the differential image (**Fig. S3D**) that is obtained by subtracting the scattering image produced by the empty nanochannel from a scattering image of the nanochannel with the biomolecule inside, $P_t - P_c \approx -2\sqrt{P_c P_m}$, can be several orders of magnitude higher than the scattering intensity collected from the biomolecule alone, had it been outside the nanochannel, $2\sqrt{P_c P_m} \gg P_m$.

1.2 Integrated optical contrast

The intensity profile of a nanochannel image (defined as the collected scattering intensity integrated along the *y*-axis) is constant along the *x*-axis and can be written as $I_c(x) = cI_o\sigma_c$. The intensity profile of the differential image contains an intensity modulation stemming from the biomolecule that resides inside the nanochannel (**Fig. S3E**). The integrated optical contrast (*iOC*) that we here define as

$$iOC = \int_{x=0}^{L} \frac{I_t(x) - I_c(x)}{I_c(x)} dx,$$

Equation 13

represents an integrated value of the intensity profile of the differential image normalized by the intensity profile of the empty nanochannel. As $\int_{x=0}^{L} I_t(x) = cLI_o\sigma_t$, Equation 13 can be written as

$$iOC = L\left(\frac{\sigma_t}{\sigma_c} - 1\right)$$

Equation 14

or, by using Equation 5, Equation 10, and Equation 11, as

$$iOC = 2m\frac{\alpha_m}{\alpha_c} + L\left(m\frac{\alpha_m}{\alpha_c}\right)^2.$$

Equation 15

For small biomolecules ($\alpha_m \ll L\alpha_c$), this can be simplified to

$$iOC = 2m\frac{\alpha_m}{\alpha_c}.$$

Equation 16

that can be expressed in terms of the refractive indexes and the cross-sectional area of a nanochannel using Equation 6 as

$$iOC = \frac{\bar{n}}{A} \cdot \alpha_m,$$

Equation 17

where

$$\bar{n}^{TE} = 2n_i^2/(n_i^2 - n_o^2)$$

Equation 18

for TE polarization and

$$\bar{n}^{TM} = (n_i^2 + n_o^2)/(n_i^2 - n_o^2)$$

Equation 19

for TM polarization.

Here, it has to be noted that the intensity profile of the nanochannel can be modulated by the profile of the incident beam (I_0 is not constant along the nanochannel). Nevertheless, the normalization by the intensity profile of the empty nanochannel from the same spot, as suggested in Equation 13, corrects for that. Moreover, as the biomolecule moves, the created image is altered by the motion blur resulting from time-averaging within one frame. However, as *iOC* represents the integrated value over the length of the nanochannel (**Fig. S3E**, Equation 13), it is not dependent on the shape of the intensity modulation stemming from the biomolecule and thus it is not altered by the motion blur. Moreover, *iOC* does not vary with the distance of the nanochannel from the focal plane, the wavelength, or NA of the imaging system. All of the aforementioned facts thus guarantee a high robustness and high precision of the measurement.

In order to confirm the conclusions of the analytical theory presented above, we have again carried out FDTD simulations. Compared to the model presented in the previous chapter, the size of the simulation cell was fixed to $3 \times 3 \times 0.5$ μm^3 and near-field data collected in the backward direction with respect to the incident field were used to simulate the dark-field image by the following procedure. First, near field monitor data were decomposed into series of plane waves that propagate at different angles using a built-in function of the software package. Any plane waves with angles outside of the specific NA were then discarded. Finally, the remaining light was re-focused onto the image plane using the chirped Z-transform. **Fig. S3** shows corresponding examples of simulated dark-field images of a biomolecule (**Extended Data Fig. 3A**), a nanochannel (**Fig. S3B**), and a biomolecule inside a nanochannel (**Fig. S3C**). **Fig. S3D** shows a differential image obtained by subtraction of the image of the nanochannel containing a biomolecule from the image of the empty nanochannel. *iOC* was then calculated by integrating the intensity profile of the normalized differential image (**Fig. S3E**) using Equation 13.

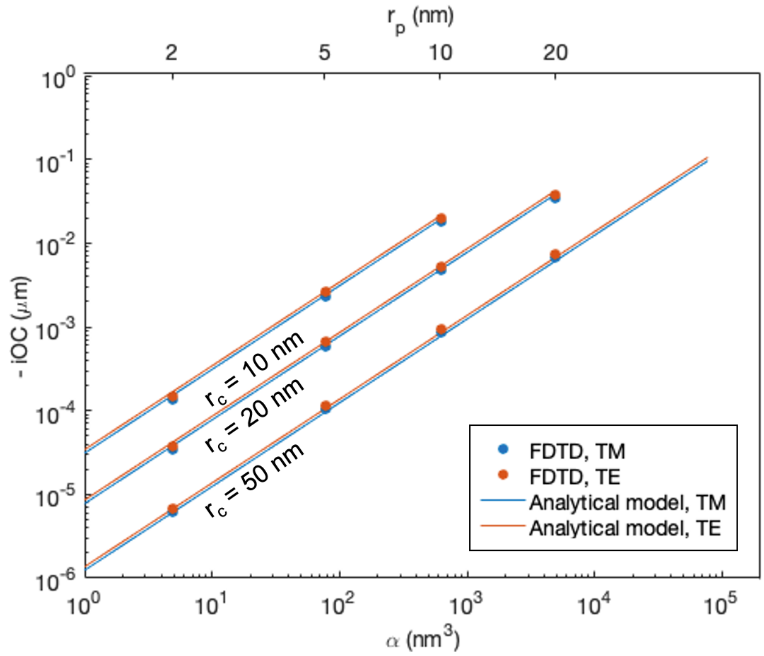

Fig. S4. Simulated iOC. *Dependency of iOC on the polarizability (bottom x-axis) and radius (upper x-axis) of a single biomolecule for nanochannels with different radius (r_c) and different polarization. The corresponding results of FDTD simulations are compared to the analytical model (Equation 17).*

The simulations were performed for a series of nanochannels with r_c = 10 – 50 nm and biomolecules with r_p = 2 – 20 nm, numerical apertures NA = 0.4 – 1, and wavelength λ = 500 – 1000 nm. **Fig. S4** shows a comparison of *iOC* calculated from FDTD simulated dark-field images and *iOC* calculated using the analytical model (Equation 17). Only the results for NA = 1 and λ = 500 nm are shown, however, the results for other combinations of NA and λ do not differ by more than 5 %. The analytical model is in excellent quantitative agreement with the FDTD simulations.

2. Light scattering of a biomolecule inside a coated nanochannel

Some NSM applications require surface modification of the channel walls, e.g., coating with a supported lipid bilayer (SLB) to prevent positively charged molecules from binding to the channel walls[4]. These modifications change the optical properties of the nanochannel and thus the optical contrast of the biomolecules inside it. Therefore, the translation of *iOC* to *MW* has to take the effect of such a coating into account when it is used.

To theoretically analyze the impact of the coating, a coated nanochannel can be described as a core-shell cylinder with one-dimensional polarizability [1]

$$\alpha_c' = A\frac{(n_s^2 - n_o^2)[n_s^2 + \nu(n_i^2 - n_s^2)(1-f)] + f n_s^2(n_i^2 - n_s^2)}{[n_o^2 + \nu(n_s^2 - n_o^2)][n_s^2 + \nu(n_i^2 - n_s^2)(1-f)] + \nu f n_s^2(n_i^2 - n_s^2)},$$

Equation 20

where n_i, n_s, n_o are the refractive indexes of the inner core, the shell, and the outside medium, respectively, A is the total cross-section area, A_i is the cross section area of the inner core, $f = A_i/A$ is the fraction of the total cross-section area occupied by the inner core, and ν is the depolarization factor ($\upsilon = 0$ for TM polarization, $\upsilon = 1/2$ for TE polarization).

The one-dimensional polarizability for TM polarization can therefore be written as

$$\alpha_c'^{TM} = A_i\left(\frac{n_i^2}{n_o^2} - 1\right) + (A - A_i)\left(\frac{n_s^2}{n_o^2} - 1\right)$$

Equation 21

and for TE polarization as

$$\alpha_c'^{TE} = \frac{2A_i\frac{n_i^2 - n_s^2}{n_i^2 + n_s^2} + 2A\frac{n_s^2 - n_o^2}{n_s^2 + n_o^2}}{1 + 2f\frac{n_i^2 - n_s^2}{n_i^2 + n_s^2}\frac{n_s^2 - n_o^2}{n_s^2 + n_o^2}},$$

Equation 22

where the second term in the denominator can be neglected for low f and small differences between the refractive indexes, $f(n_i^2 - n_s^2)(n_s^2 - n_o^2) \ll 1$.

When a biomolecule is present inside such a nanochannel, its contribution can be described in terms of increased effective permittivity of the inner core (Equation 7) and the total polarizability of a nanochannel containing a biomolecule that can then be written as

$$\alpha_t'^{TM} = A_i\left[\frac{n_i^2}{n_o^2} - 1 + \frac{n_i^2}{n_o^2}n_V\alpha_m\right] + (A - A_i)\left(\frac{n_s^2}{n_o^2} - 1\right),$$

Equation 23

$$\alpha_t'^{TE} = A_i\left[\frac{2\frac{n_i^2 - n_s^2}{n_i^2 + n_s^2} + n_V\alpha_m}{1 + \frac{n_V\alpha_m}{2}\frac{n_i^2 - n_s^2}{n_i^2 + n_s^2}}\right] + 2A\frac{n_s^2 - n_o^2}{n_s^2 + n_o^2}.$$

Equation 24

For biomolecules with low polarizability that are sparsely distributed inside the nanochannel, and for a small difference between the refractive indexes, $n_V \alpha_m (n_i^2 - n_o^2) \ll 1$, the second term in the denominator in Equation 24 can be neglected and the polarizability of the nanochannel containing the biomolecule (Equation 8 and Equation 9) can be written as

$$\alpha_t' = \alpha_c' + m\, \alpha_m / L,$$

Equation 25

where $m = 1$ for TE polarization and $m = n_i^2/n_o^2$ for TM polarization. Note that the expression for the total polarizability of a core-shell cylinder (Equation 25) corresponds to the total polarizability of a cylinder without a shell (Equation 10), and therefore, Equation 11 to Equation 17 are valid for the core-shell cylinder as well. The value of the parameter $\bar{n}$ in Equation 17 is, however, different, as it accounts for the presence of the additional layer. It can be written as

$$\bar{n}' = \bar{n} \frac{\alpha_c}{\alpha_c'} = \bar{n} \sqrt{\frac{I_c}{I_c'}}$$

Equation 26

where I_c/I_c' is the ratio between intensity of the scattered light from a cylinder with and without a coating (additional layer).

3. Polarizability of a protein

The fact that the refractive index of a protein solution increases linearly with the (mass) concentration of the protein (c) and that the specific refraction increments dn/dc have almost the same value for a large number of different proteins ($dn/dc = 0.185\ \mathrm{mL \cdot g^{-1}}$) has been pointed out by many studies[5].

Using the Clausius-Mossotti relation

$$(n^*)^2 \approx n^2\left(1 + \frac{\alpha_m n_V}{1 - \frac{\alpha_m n_V}{3}}\right),$$

Equation 27

that describes the relation between the polarizability of biomolecules present in solution (α_m), volume density of the biomolecules (n_V), refractive index of the medium in which the biomolecules are present (n), and the (effective) refractive index of the solution n^*, the relation between refractive index increment (dn/dc), molecular weight (MW), and the polarizability of a biomolecule normalized by MW can therefore be expressed as

$$\frac{\alpha_m}{MW} = \frac{2}{nN_A}\frac{dn^*}{dc} = 0.46\frac{\text{Å}^3}{\text{Da}},$$

Equation 28

where $\alpha_m n_V \ll 1$ was assumed, $n = 1.33$ RIU (water), $c = MW \cdot n_V/N_A$, and $N_A = 6.022\ \times 10^{23}$ is Avogadro's number.

Interestingly, the same value of α_m/MW can be derived from a calculation of the properties over 171,729 different proteins presented by Young et al. that determined the mean specific volume of a protein (defined as volume per mass) as V_{sp} = 0.7446 mL g^{-1} and the mean refractive index of a protein as n_m = 1.5867 RIU. Using the electrostatic approximation (Equation 6), the polarizability of a protein can then be expressed as

$$\frac{\alpha_m}{MW} = \frac{3V_{sp}}{N_A}\frac{n_m^2 - n_i^2}{n_m^2 + 2n_i^2} = 0.46\frac{\text{Å}^3}{\text{Da}}.$$

Equation 29

Since *iOC* is linearly proportional to the polarizability of a biomolecule (Equation 17), and since the polarizability of a biomolecule is linearly proportional to the molecular mass (Equation 28 and Equation 29), the molecular mass of a biomolecule inside a nanochannel can indeed be directly determined from *iOC* as

$$MW = iOC \cdot \frac{A}{\bar{n}a},$$

Equation 30

where $a = \alpha_m/MW = 0.46\ \text{Å}^3 \cdot \mathrm{Da^{-1}}$ and $\bar{n}, A$ are constants related to the properties of the nanochannels that can be determined prior to the measurements. In this study, the parameter A was determined from SEM images of the nanochannel cross section (**Extended Data Fig. 3**).

Parameter $\bar{n} = -10.25$ was calculated from Equation 18 and Equation 19, where $n_i = 1.33$ RIU (water), $n_o = 1.46$ RIU (SiO_2) and even representation of TE and TM polarization was assumed, $\bar{n} = 0.5(\bar{n}^{TE} + \bar{n}^{TM})$. For nanochannels coated with a lipid bilayer, a modified parameter $\bar{n}' = \bar{n}\sqrt{I_c/I_c'}$ was used (Equation 26), where $I_c/I_c' = 1.7$ for Channel VI was determined from the intensity of scattered light from the nanochannel before and after the deposition of the lipid bilayer.

4. Error estimate of size determination due to partial-slip boundary condition

The size of individual particles is related to the diffusion constant through the Stokes-Einstein relation corrected for the hindrance effect due to the small nanochannel size as

$$R_s = K \cdot \frac{k_B T}{6\pi\eta D},$$

Equation 31

where K is the hindrance factor[6], $K = (1 + 9\lambda \cdot \ln\lambda/8 - 1.56\lambda + 0.53\lambda^2 + 1.92\lambda^3 - 2.81\lambda^4 + 0.27\lambda^5 + 1.1\lambda^6 - 0.44\lambda^7)/(1-\lambda)^2$, and $\lambda = R_s/r$, $r = \sqrt{A/\pi}$ is the radius of a circle defined by an area A. This expression assumes that the flow velocity at the boundary of the nanochannel vanishes, known as the "no-slip" condition. Recently, it was demonstrated[7] that SLB do not obey the no-slip condition. This can be taken into account by introducing a "slip length" l, which denotes the distance below the surface of the bilayer at which the flow profile vanishes by extrapolating the flow profile. For bilayers, this was found to be around l = 5 nm. In order to account for this effect, we first note that the hindrance factor K is obtained by averaging the influence of the hindrance on the nanoparticle diffusion over a cross section of the nanochannel,

$$K(r) = 2/(r-R)^2 \int_0^{r-R} D_{||}(r-\rho; R)/D_\infty \, \rho \, \mathrm{d}\rho$$

Equation 32

Where D_∞ is the bulk diffusivity, R is the radius of the particle, $D_{||}$ is the diffusion constant parallel to the boundary[7]

$$D_{||}(l;R) = 1 - \frac{9}{16}\left(\frac{R}{l}\right) + \frac{1}{8}\left(\frac{R}{l}\right)^3 - \frac{45}{256}\left(\frac{R}{l}\right)^4 - \frac{1}{16}\left(\frac{R}{l}\right)^5,$$

Equation 33

To lowest order, the effect of the slip length can be taken into account by extending the dimension of the channel to $r' = r + l$, while taking into account that the channel wall at $\rho = r$ still constitutes the physical boundary. The integral boundaries therefore do not change, but the integrand changes to $D_{||}(r + l - \rho; R)/D_\infty$. The effective hindrance factor can then be approximated as

$$\widetilde{K}(r) \approx K(r+l) - \left(\frac{l}{r}\right) D_{||}(l;R)/D_\infty.$$

Equation 34

The resulting effective hindrance factor relevant for Channel V (A_V = 225 × 200 nm^2) is shown as a function of particle radius in **Fig. S5**, normalized to the hindrance factor assuming no-slip condition, $\widetilde{K}/K$. For particles up to 70 nm diameter (range of the BNPs detected in Channel V), it ranges between 0.96 – 1.01. This suggests that the estimated error in size determination of BNPs presented in the context of the analysis of the conditioned cell culture medium (**Fig. 4C-F**) is lower than 4% and therefore the effect of the partial-slip boundary condition can be safely ignored.

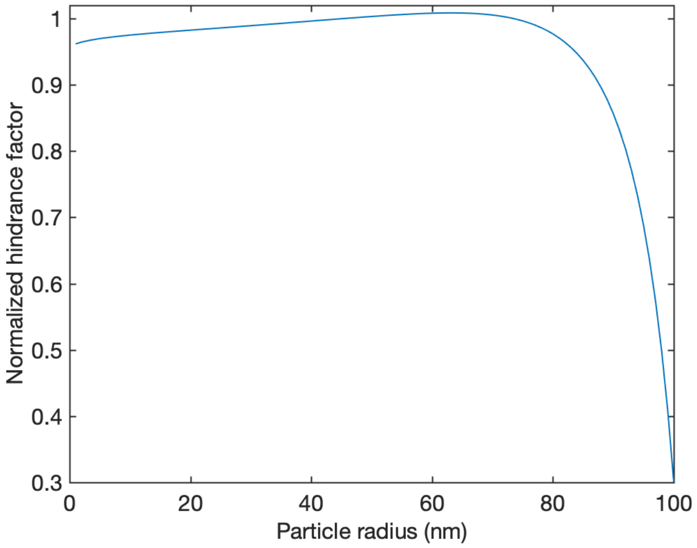

Fig. S5. Normalized hindrance factor ($\widetilde{K}/K$) *as a function of particle radius for Channel V (A_V = 225 × 200 nm^2) that takes into account the partial-slip boundary condition relevant for SLB.*

5. Removal of the background

To visualize the biomolecules and their trajectory inside a nanochannel in the form of kymographs (**Fig. 2**), the data acquired by the CMOS camera were processed using the following procedure.

In the first step, the raw images of the nanochannel in every frame were integrated along the short axis of the nanochannel and averaged using convolution with a Gaussian function with 18 pixels width. This step results in time series of an intensity profile of the nanochannel, $I_i(x)$, where *i* stands for *i*-th time frame and *x* is a spatial coordinate.

In the second step, the nanochannel's profile was corrected for spatial and intensity instabilities. At first, to correct for the spatial instabilities (vibrations of the system), the image profile in every i-th step spatially shifted by Δ_i was estimated using spline interpolation (Matlab). After that, to correct for the intensity instabilities, the normalized differential image profile ($\overline{I_\iota}(x)$) was calculated as

$$\overline{I_\iota}(x) = \frac{I_i(x+\Delta_i)}{I_{i+1}(x)} - 1,$$

Equation 35

from which the low frequency modulation was filtered using

$$\overline{I_i^*}(x) = \frac{\overline{I_\iota}(x)}{\overline{I_\iota}(x) * h(x)},$$

Equation 36

where the denominator in Equation 36 stands for the convolution of $\overline{I_\iota}(x)$ with a step function $h(x)$ with width $A_x = 200$ pixels, defined as

$$h(x) = \frac{1}{A_x}, \ |x| \leq A_x/2$$
$$h(x) = 0, \ |x| > A_x/2$$

Equation 37

The spatial shifts in every i-th step, Δ_i, were found in an iterative procedure based on the Newton-Raphson method that finds a minimum of the function

$$f(\Delta_i) = \int_{x=0}^{L_{fow}} |\overline{I_i^*}(x+\Delta_i)| dx,$$

Equation 38

where L_{fow} is the length of the field of view. More specifically, a refined *n*-th guess, Δ_i^n, is estimated from two previous guesses Δ_i^{n-1} and Δ_i^{n-2}, as

$$\Delta_i^n = \frac{\sum_x w(x) \cdot \delta(x)}{\sum_x w(x)}$$

Equation 39

$$\delta(x) = \Delta_i^{n-2} - \frac{(\Delta_i^{n-1} - \Delta_i^{n-2}) \cdot \overline{I_i^*}(x+\Delta_i^{n-2})}{\overline{I_i^*}(x+\Delta_i^{n-1}) - \overline{I_i^*}(x+\Delta_i^{n-2})}$$

Equation 40

where $w(x) = \left|\overline{I_i^*}(x + \Delta_i^{n+1})^* - \overline{I_i^*}(x + \Delta_i^n)\right|$ is the weight function. The iterative process described in Equation 39 and Equation 40 is repeated until a minimal value of $f(\Delta_i)$ (Equation 38) is reached with sufficient precision.
The time series of corrected nanochannel profiles, $I_i^*(x)$, is then calculated from $\overline{I_i^*}(x)$, as

$$I_{i+1}^*(x) = \frac{I_i^*(x + \Delta_i)}{\overline{I_i}(x) + 1},$$

Equation 41

and $I_1^*(x) = I_1(x)$.

In the third step, the background (intensity profile of an empty nanochannel), I_i^b, is estimated from corrected image profiles, as

$$I_i^b(x_j) = \text{median}\left(\hat{I}(x_j)\right), \hat{I} = \{I_{i-A_t/2} \quad \cdots \quad I_{i+A_t/2}\},$$

Equation 42

where a window of size A_t = 100 frames was used, whose size was truncated at the endpoints. Kymographs are then obtained by subtracting the background followed by normalization by the background, as

$$I_i^k(x) = \frac{I_i^*(x) - I_i^b(x)}{I_i^b(x)}.$$

Equation 43

In the fourth step, the positions of the biomolecule are found using the particle tracking algorithm, described in detail in SI section "Particle tracking" and a matrix $M_i(x)$ was created for which $M_i(x) = I_i^k(x)$ when the *i-th* time frame and *x* position correspond to a found particle, and $M_i(x) = 0$ otherwise.
In the last step, all the four steps are repeated, but the intensities that were identified by the particle tracking algorithm as responses corresponding to biomolecules are excluded from the process by diving the time series of an intensity profile of the nanochannel, $I_i(x)$, by the matrix $M_i(x)$. The last step is repeated iteratively until convergence is achieved, i.e., the difference between $I_i^k(x)$ derived in the subsequent iteration steps is not larger than the standard deviation of I_k.

6. Particle tracking

To find the positions of a biomolecule and link them to a trajectory, we have used the particle tracking algorithm described in [8]. In order to improve the performance for biomolecules with low *iOC*, we have modified some parts of the algorithm, as described below.
To detect possible biomolecules, all the minima in every time frame of the kymograph (I^k) were found and their zero order intensity moments (m) and positions (x) were determined using a procedure described in [8]. In the next step, the found minima were used to create all possible short trajectories with temporal length of 8 frames for which the difference of the positions between two subsequent frames were lower than $3\sqrt{2\Delta t D_{limit}}$, where Δt is the time difference between two subsequent frames and D_{limit} is an optional parameter setting the highest diffusivity of a biomolecule that would be found by the algorithm. In all presented cases we have used a $D_{limit} = 50$ µm²/s. The mean value of m ($\overline{m(t_j)}$), standard deviation of m ($\sigma_m(t_j)$), and the diffusivity ($D(t_j)$) of every trajectory t_j that is composed of 8 found minima (possible biomolecules, p_i) is calculated according to

$$\overline{m(t_j)} = \frac{1}{8}\sum_{i=1}^{8} m(p_i),$$

$$\sigma_m(t_j) = \sqrt{\frac{1}{7}\sum_{i=1}^{8}\left(m(p_i) - \overline{m(t_j)}\right)^2}$$

$$D(t_j) = \frac{1}{7}\sum_{i=1}^{7}\frac{\left(x(p_{i+1}) - x(p_i)\right)^2}{2\Delta t}$$

Equation 44

To evaluate the characteristics of the noise, we followed the same procedure for a kymograph with inversed intensity ($-I^k$), i.e., we find all minima, create all possible short trajectories (t_j^n) and calculate the mean value and the standard deviation of m that correspond to the noise ($\overline{m(t_j^n)}, \sigma_m(t_j^n)$). From the set of the values $\overline{m(t_j^n)}$ we determine its mean value and standard deviation, and define a limiting value $\overline{m_{limit}} = \text{mean}\left(\overline{m(t_j^n)}\right) - 4\text{std}\left(\overline{m(t_j^n)}\right)$. In a similar fashion, from a set of values $\sigma_m(t_j^n)$ we determine its mean value and standard deviation and define a limiting value $\sigma_m^{limit} = \text{mean}\left(\sigma_m(t_j^n)\right) + 4\text{std}(\sigma_m(t_j^n))$. All found possible trajectories for which $\overline{m(t_j)} > \overline{m_{limit}}$ and $\sigma_m(t_j) > \sigma_m^{limit}$ are then considered to be noise and are discarded. All the remaining trajectories (with temporal length of 8 frames) are combined into all possible trajectories of longer temporal lengths and their cost function is calculated as

$$\phi_j = D(t_j) \cdot \sigma_m^2(t_j).$$

Equation 45

The complete set of single biomolecule trajectories is then selected from longest trajectories with lowest cost function while assuming that every found minima is assigned to only one biomolecule. Trajectories that were shorter than 40 frames were considered to correspond to noise and thus discarded.

In the last step of the algorithm, parts of the trajectories that correspond to the biomolecules that are bound to the surface (not diffusing) are found using the following procedure. We assume that if the biomolecule is bound to the surface (does not change its position), the standard deviation of the found position, corresponds to the (yet unknown) localization error, $\sigma_x = \delta$, that is related to the noise in the kymographs. The standard deviation of the spatial displacement in time then corresponds to $\sigma_{\Delta x} = \sqrt{2}\delta$. Therefore, the ratio of the two standard deviations corresponds to $\sigma_x/\sigma_{\Delta x} = 1/\sqrt{2}$. On the other hand, if the biomolecule is diffusing, the standard deviation of the displacement in time corresponds to $\sigma_{\Delta x} = 2D\Delta t + \delta$, where D is the diffusivity of a biomolecule and Δt is the time difference between two subsequent frames, and the standard deviation of the position corresponds to $\sigma_x = D\Delta t\sqrt{2N} + \sqrt{2}\delta$ where N is the number of frames. Therefore, the ratio of the two standard deviations then increases with number of frames and for $N \gg \delta/D\Delta t$ corresponds to $\sigma_x/\sigma_{\Delta x} = \sqrt{2N}/2$. It can be seen that $\sigma_x/\sigma_{\Delta x}$ differs between the two states of the biomolecule (diffusing or bound) and can thus be used as merit to distinguish those two different states. Therefore, for every part of a trajectory with temporal length of $N = 30$ frames, $\sigma_x/\sigma_{\Delta x}$ is calculated and if the derived value is lower than the threshold limit 0.9, the part of the trajectory is assigned to a biomolecule that is bound to the surface and it is discarded from the further analysis.

The integrated optical contrast of every found minimum in the kymograph assigned to a biomolecule can be determined as $iOC_n = \sqrt{\pi}ab$ where the values a and b correspond to the values found by the least-square fitting of the gauss function $a\exp(-(x - x_i)^2/b^2)$ (*fit* function from Matlab library) where x_i corresponds to the position of the biomolecule determined by the particle tracking algorithm. Each single biomolecule is then represented by N values of iOC_n. iOC pertaining to a single trajectory is then the mean value of iOC_n. Alternatively, the values of I^k from the interval of positions $\langle x_i - w_{DLS}, x_i + w_{DLS}\rangle$ can be averaged over time frames corresponding to one trajectory that results in the time-averaged intensity profile of spatial length of $2w_{DLS}$. iOC can then be determined as $iOC = \sqrt{\pi}ab$ where the values a and b correspond to the values found by the least-square fitting of the gauss function $aexp(-x^2/b^2)$. Both of these approaches resulted in comparable results, however, the second one lead to an improved processing speed.

The diffusivity was obtained from the statistical analysis of the spatial displacement in time determined by the particle tracking algorithm as[9]

$$D = \overline{(\Delta x_n)^2}/2\Delta t + \overline{\Delta x_n \Delta x_{n+1}}/\Delta t.$$

Equation 46

It has to be noted, that the second factor corrects for both motion blur and the localization error[9].

7. Machine learning (ML) analysis

To track and analyze single biomolecules within the nanochannels using a method that is independent from the standard analysis (SA) presented in the SI sections "Removal of the background" and "Particle tracking" and therefore validate its results, we implemented a machine-learning (ML) analysis workflow[10] whose results are summarized in **Extended Data Fig. 4**. It is comprised of five parts:

1. Pre-processing of raw image data into kymographs.
2. Image segmentation using a U-net neural-network[11] to identify the position of the single biomolecules.
3. Object detection using the YOLOv3 algorithm[12] to identify the trajectories of the single biomolecules.
4. Property calculation using a custom fully convolutional neural network (FCNN) with residual connections to determine the properties of the single biomolecules (iOC and D).
5. Conversion from iOC to MW and from D to R_s.

All intermediate results of the ML algorithm are described in the sub-sections below, and the whole pipeline is summarized in **Fig. S6**.

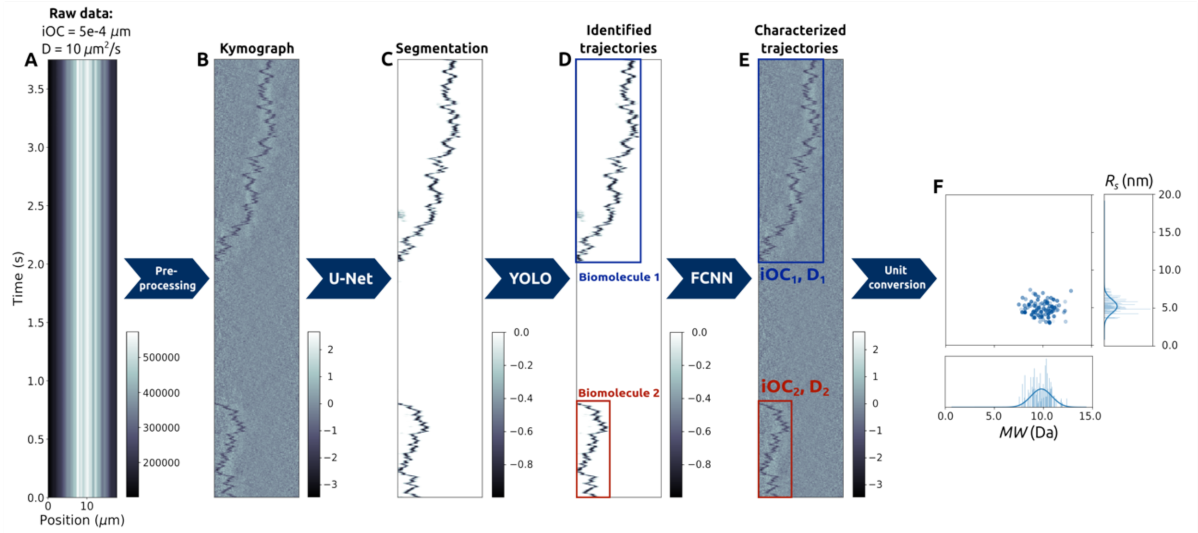

***Fig. S6. Machine learning (ML) analysis workflow. (A)** Raw image data simulated for a single biomolecule with optical contrast $iOC = 5 \cdot 10^{-4}\ \mu m$ and diffusivity $D = 10\ \mu m^2/s$. **(B)** Preprocessed kymograph with removed background. **(C)** Segmented image where the particle positions are detected using a U-net. **(D)** Single biomolecule trajectory identification using the YOLOv3 neural network. **(E)** Property calculation (iOC and D) using a custom fully connected neural network (FCNN). **(F)** Unit conversion from iOC to molecular weight MW and from D to hydrodynamic radius R_s plotted in a 2D histogram for illustration purposes.*

1. Data pre-processing from raw data to kymograph

The raw image data (**Fig. S6A**) was pre-processed to transform it into kymographs (**Fig. S6B**). First, the intensity of the raw CMOS image data was normalized according to

$$\bar{I}(x,t) = \frac{I(x,t) - \langle I(x,t)\rangle}{\langle(I(x,t)\rangle}$$

where $I(x,t)$ is the intensity at position x and time-frame t, and $\langle I(x,t)\rangle$ represents its time average. Second, a low-pass-filtered version of $\bar{I}(x,t)$ was calculated by using two normalized sliding windows of sizes 200×1 and 1×200, and subtracted from $\bar{I}(x,t)$. Finally, to obtain the kymographs used to calculate iOC, the resulting image was down-sampled by a factor of 4 in the length dimension through mean pooling. To obtain the kymographs used to calculate D, the image was instead normalized by its standard deviation before being down-sampled by a factor of 4 in the length dimension through mean pooling.

2. Image segmentation with U-net

Image segmentation is used to make the value of each pixel of a given image more representative of a property of interest. In our case, we transform a kymograph where the value of each pixel represents intensity (**Fig. S6B**), to a segmented image where the value of each pixel represents the probability P_{b} of the existence of a biomolecule in that location and time (**Fig. S6C**). We perform this transformation using a U-net[11] implemented using the Python software package DeepTrack 2.0[10]. The details of the U-net architecture are shown in **Fig. S7A**. The U-net was trained as part of a conditional Generative Adversarial Network (GAN)[13]. This GAN consists of two neural networks (**Fig. S7B**): a *generator* network (the U-net), which generates image segmentations based on input kymographs; and a *discriminator* neural network, which attempts to figure out whether the generated segmentations are the real trajectories of single biomolecules in the input kymographs. During the training process, the two networks compete against each other, so that the generator generates increasingly realistic trajectories, and the discriminator learns increasingly subtle ways to distinguish generated from real trajectories. Using this GAN for training was essential to achieve the required accuracy especially for smaller biomolecules, for which the low signal-to-noise ratio often results in trajectories being entirely overpowered by noise in several subsequent time frames.

The U-net was trained on simulated kymographs (generated as described in subsection 1 and pre-processed as described in subsection 1) for which the corresponding ground-truth single biomolecule trajectory is known (using the ADAM optimizer[14] with an exponentially decaying learning rate of 10^{-6}, a decay rate of 0.9, and 50 decay steps). The U-net was trained using 300,000 simulated kymographs with particle trajectories in the ranges $1 \cdot 10^{-5}$ µm $\leq iOC \leq 3 \cdot 10^{-3}$, $1 \leq \mathrm{D} \leq 100\ \mu\mathrm{m}^2/\mathrm{s}$. The input data during training are simulated images (kymographs) of size 128×2048 with a random number of trajectories and of output segmented images of equivalent size. The model is also train-validated every 120 epochs (=~30000 simulated kymographs) against 150 simulated kymographs (of size 128×512, 128×1024, 128×2048) with experimentally measured channel noise, using an 80-20 train-validation split.

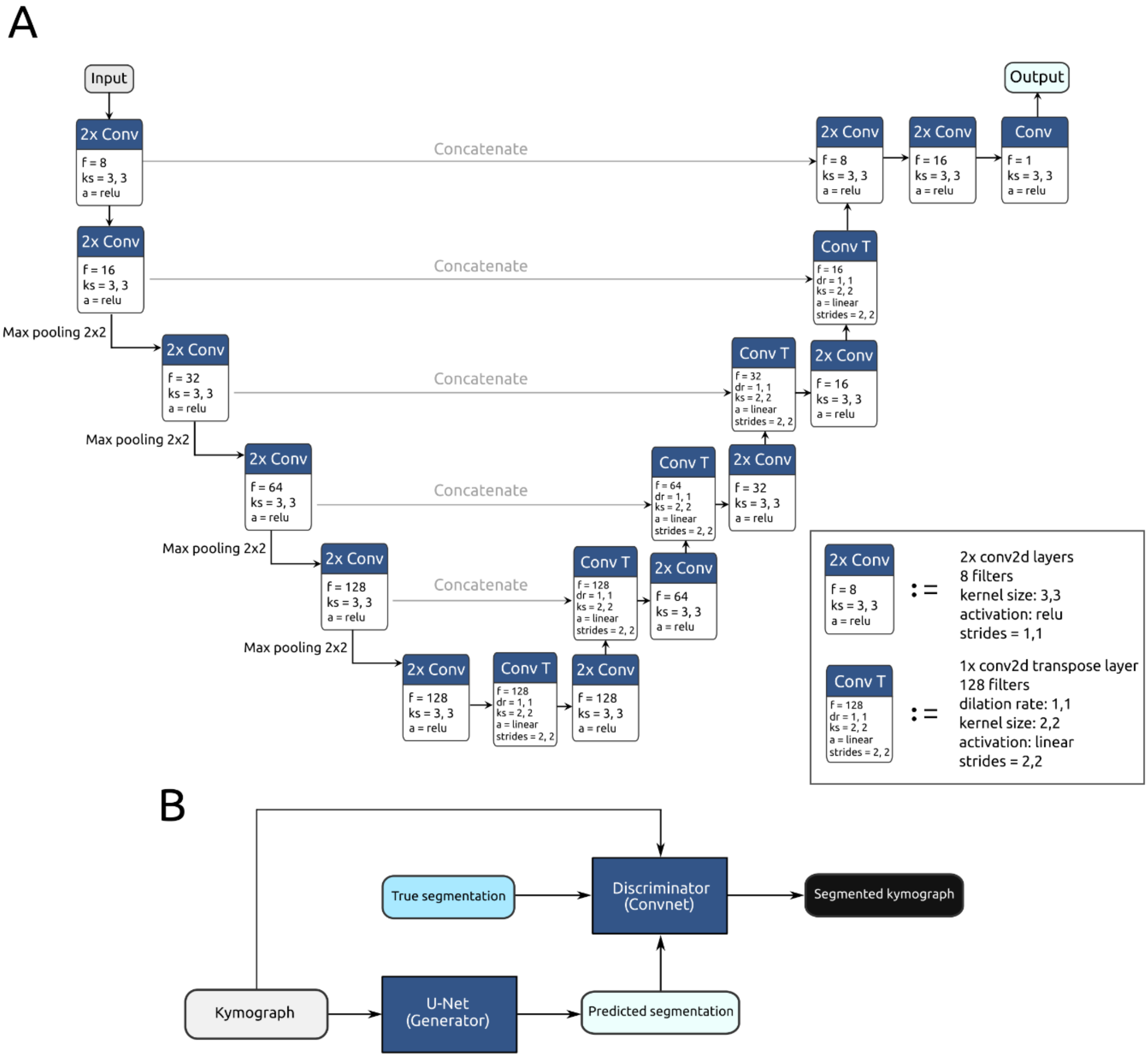

Fig. S7. U-net for single biomolecule segmentation. (A) *The U-net*[11] *consists of a series of contraction convolutional layers, a bottleneck, and a series of expansion convolutional layers, as well as a series of skip connections between corresponding contraction and expansion convolutional layers to ensure that information learnt during contraction is not lost at the bottleneck. Each "2× Conv" box represents a convolutional block corresponding to 2 convolutional layers in sequence, and each "Conv T" box represents a single convolutional transpose layer as exemplified in the legend. Here, f is the number of filters in each block, ks is the kernel size,* a *is the activation function, and* dr *is the dilation rate. The network is visualized with Netron*[15]*.* ***(B)*** *Block diagram of the conditional GAN training environment, where kymographs are fed both to a U-net generator network, which predicts a corresponding segmentation, and a Convnet (convolutional network) discriminator network, which takes both original kymographs and true segmentations as input to determine whether the predicted segmentation is correct. A basic Convnet consisting of 5 convolutional layers of size 4×4 and stride 1 connected to a single dense layer is used as discriminator network*[16]*.*

3. Trajectory identification with YOLOv3

As third stage of our analysis, we implemented a YOLOv3[12] (**Fig. S6D**) object detection algorithm in PyTorch[17] to identify single biomolecule trajectories by separating a given kymograph into smaller ones, each corresponding to a single biomolecule trajectory. If a kymograph cannot be separated in this manner (for instance, as a result of two biomolecule trajectories strongly overlapping with each other), the YOLO algorithm instead counts the number of inseparable single biomolecule trajectories in the given kymograph and outputs this value. The network architecture behind this algorithm is visualized in **Fig. S8**.

This algorithm analyses the segmented images obtained by the single-biomolecule-tracking U-net described in subsection 2 to fit a minimally-dimensioned rectangular bounding box around each separate single biomolecule trajectory. If the minimal bounding boxes of two single biomolecule trajectories overlap more than the set threshold $b_t = 60\%$, their bounding boxes are merged to a minimal rectangular bounding box around both of them that is labelled as containing $two\ biomolecules$. If three or more trajectories overlap, their bounding boxes are merged and labelled as $three + biomolecules$.

The YOLOv3 algorithm was trained on simulated kymographs, generated in the same way as for the U-net (described in subsection 1 above), but where the input is a perfectly segmented kymograph and the outputs are minimally sized bounding boxes around each separate trajectory. Trajectories are considered separate in the training data if they are more than $T/16$ frames apart, where T is the total amount of frames in the kymograph. Images are continuously generated during training, such that each subsequent image is entirely unique and the risk of overtraining is avoided. The algorithm was trained using approximately 2 million such unique segmented images, where each image was generated with a diffusion in the range $D \approx 1 \text{ to } 100\ \mu\text{m}^2/s$ (the optical contrast is not relevant here because we employ perfectly segmented simulated images) with the standard YOLO error function[12] using the ADAM optimizer[14] with a learning rate of 0.001. The input data during training are simulated images (segmented kymographs) of size 128×8192, down-sampled to 128×128 to improve performance, and the output is a list of YOLO-labels containing class, position, occurrence probability and class probability of each trajectory in the input image.

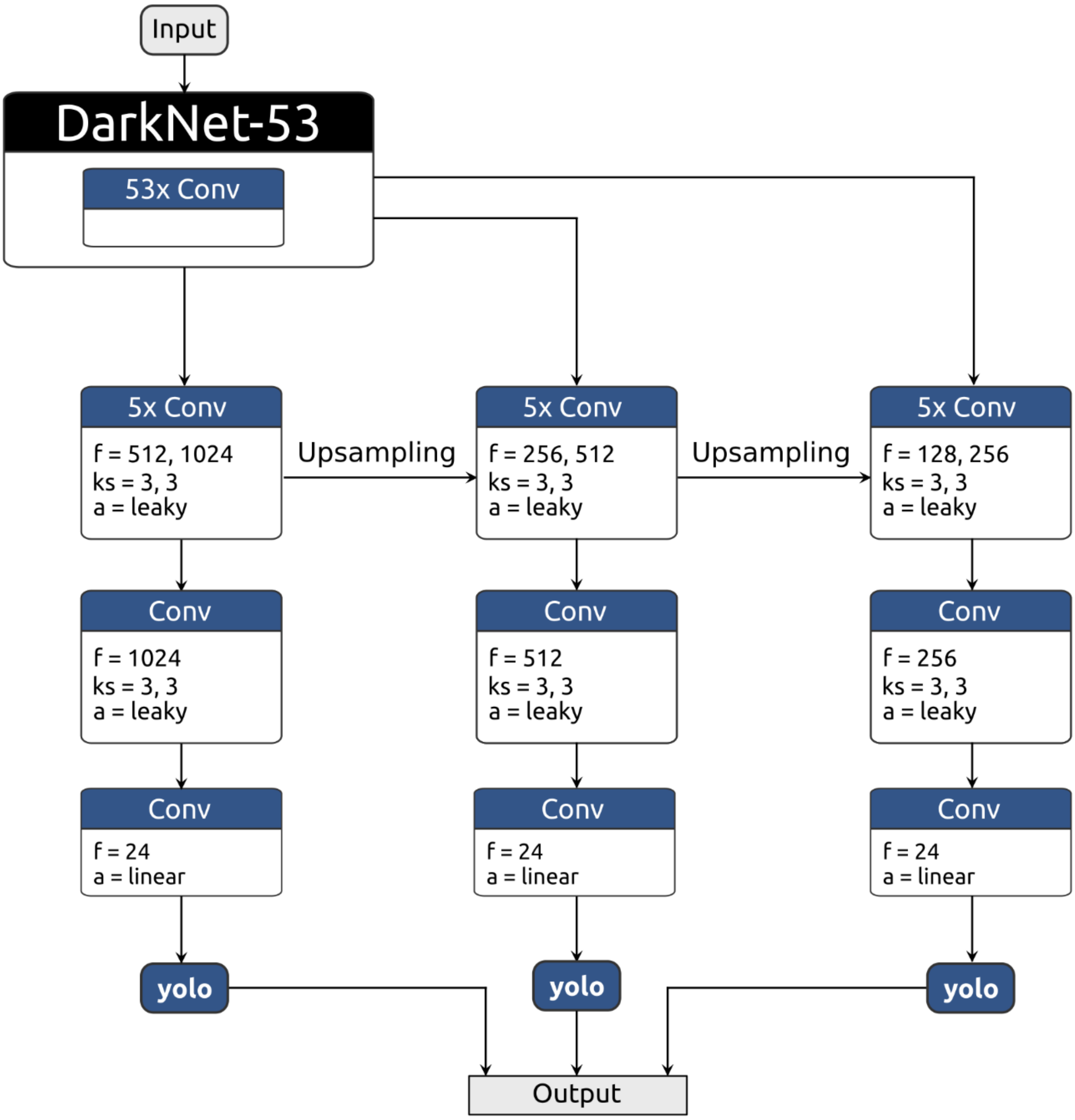

Fig. S8. YOLOv3 for trajectory identification. *The YOLO algorithm is built upon a DarkNet-53 backbone*[18]*, which consists of a neural network architecture comprised of 53 convolutional layers. This backbone feeds into three necks, each consisting of a sequence of 5 convolutional layers, which in turn feed into corresponding heads, which output bounding boxes at three different scales through YOLO detection layers*[12]*. Each neck is up-sampled and fed into its neighboring neck to make sure the last yolo detection layer benefits from information collected at every previous stage. Here, "Conv" represents a single convolutional layer, f is the number of filters in each block,* ks *is the kernel size, and* a *is the activation function. The network is visualized with Netron*[15]*.*

4. Property calculation with FCNN

To calculate iOC and D for each single biomolecule trajectory (**Fig. S6E**), we use a custom neural network architecture, which consists of a FCNN connected with residual blocks. The architecture itself is a sequence of convolutional layers followed by a max pooling layer, including short skip connections to preserve information, as shown in **Fig. S9**. The full architecture consists of seven of these blocks, culminating in a *head* module where the algorithm produces slightly different outputs depending on whether we are calculating iOC or D.

To calculate the iOC, the *head* of the neural network returns three outputs: (1) The raw output I_{matrix} from the last convolutional layer of the neural network. (2) The *mask,* which is a down-sampled representation of the original kymograph normalized by its pixel sum. This effectively gives us a weighted matrix, where each matrix element is the probability that the corresponding down-sampled region in the kymograph contains a biomolecule trajectory. (3) The calculated iOC for each kymograph, obtained by multiplying I_{matrix} by the *mask* to produce a value of iOC for each pixel in the down-sampled kymograph weighted by the probability of said pixel containing a trajectory, and finally summing over the entire down-sampled image to obtain a single mean value of iOC for the entire kymograph.

Calculating D works as for iOC, except that we use the down-sampled U-net biomolecule-tracking algorithm's pre-segmented image as the *mask*, instead of re-calculating it using the FCNN. This is done for two reasons: because the intensity network cannot be trained on segmented images since iOC is entirely dependent on the noise in an image, and because the diffusion calculation is inherently sensitive to the quality of the single biomolecule trajectory tracking so that a more powerful segmentation model is needed.

To train the intensity- and diffusivity-calculating FCNN models, we employed a curriculum learning scheme with intermittent checkpoints to be used for later ensemble modelling prediction. Specifically, the intensity-calculating model was initially trained only on a narrow range of high *iOC* trajectories, representing the highest SNR and in principle easiest case for the model to begin learning correlations, and then slowly curriculum-learned down to the lowest range of relevant *i*OC values. In each narrow range of *iOC* values, checkpoint models which are more accurate in that particular narrow range of values are saved separately. Upon model inference, an initial prediction is made with a model trained on the entire range of *iOC* values with the scheme described above, and then a second model trained on the narrower range of values makes a second prediction on the same trajectory to achieve higher accuracy. The process is equivalent for diffusivity, with the difference being that the range of D values being trained on increases rather than decreases during curriculum learning.

These were all trained using the ADAM optimizer[14] with a learning rate of 0.0001 on approximately 300,000 simulated kymographs in the same range as those employed for the U-net. The input during training of the FCNNs are simulated images (kymographs) of size 128×2048 with a single particle trajectory, and the output is a single value of either *iOC* or D of said trajectory, as well as the *mask,* which is a learnt downsampled representation of the original kymograph.

We note here that the ensemble modelling technique adds yet another barrier against false signal detection, since if the epistemic uncertainty inherent in the data is high, which may be the case for very short trajectories or regions of unusually high noise, the two predictions will differ significantly and hence the prediction should be discarded. Of course, as is common in ensemble modelling [19], one can implement an even larger and finer-grained ensemble for even higher prediction accuracy and more accurate uncertainty estimation.

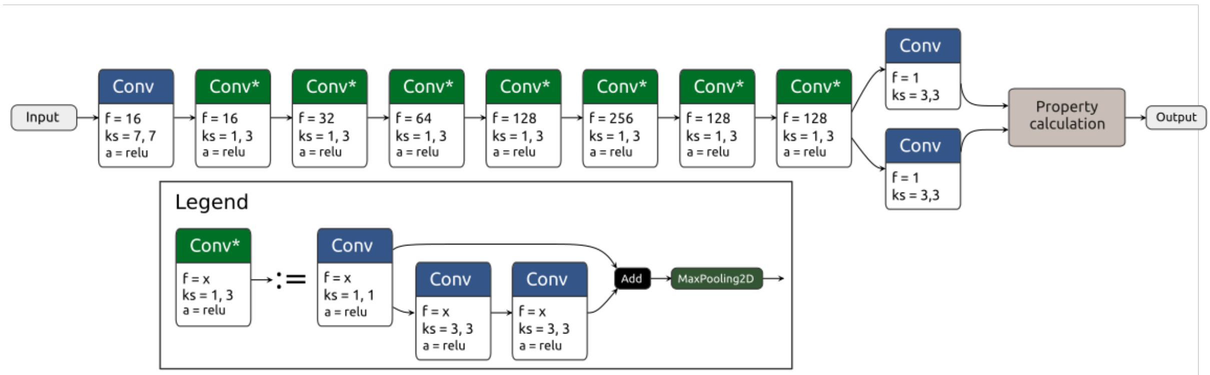

Fig. S9. FCNN for property calculation. *The custom FCNN architecture is comprised of "Conv" blocks, which are singular convolutional layers, and a sequence of "Conv*" blocks, which consist of a sequence of convolutional layers followed by a max pooling layer and a skip connection, as exemplified in the legend. This sequence culminates into a custom property layer that calculates* iOC *and* D. *Here,* f *is the number of filters in each block,* ks *is the kernel size, and* a *is the activation function. The network is visualized with Netron*[15].

5. ML analysis results

In **Extended Data Fig. 4** we show the results using the ML analysis, which are in agreement with the results obtained using the SA analysis presented in **Fig. 3**. Note that, since the ML analysis retrieves longer trajectories that the SA analysis maximizing the available data for each data point, this results in fewer data points for the ML results than for the SA results, even though the total amount of data is the same for both methods. Since the ML analysis is completely independent from the SA analysis, the agreement between these two analyses further strengthens the confidence that these results are correct. The ML method is fully automatized and operates 1-2 orders of magnitude faster than the SA method.

6. ML analysis of cell conditioned medium

To analyze the cell conditioned media, the overall ML algorithm is the same as described above, with the exception that the YOLO is replaced with a thresholding function on the segmented kymographs and the U-net/FCNN models are trained in slightly different schemes. In general, YOLOv3 struggles to identify object boundaries in situations where several small objects are in very close proximity or even overlap, as has been shown consistently with, e.g., flocks of birds or other small animals[20]. As a result of the very high concentration of lipoprotein trajectories in the segmented kymographs, this limitation adversely affects EV analysis as lipoprotein trajectories may inadvertently be included in the bounding boxes of otherwise isolated EV trajectories and lead to inaccurate results. To avoid this issue, we implemented a simple thresholding function, which works by first identifying the starting point of each new trajectory (under flow) in a segmented kymograph (i.e., time t_0 where $P_\mathrm{b} > threshold$ at $x_0 = 0$). Beginning with the first trajectory, we investigate all points in a square region of size $d_t \times d_t$ at (x_0, t_0) where $P_\mathrm{b} > threshold$. If none of these points are of equal t and unequal x, or vice versa, we consider the box to only contain a single trajectory, and the process is repeated in a box of size $d_t \times d_t$ at (x_i, t_i) where $x_i = x_0 + id_t$, $t_i = \mathrm{t}(x_i)$ in iteration i. This forms a sequence of boxes $[b_0, b_1, \dots, b_i]$ which are considered to contain a single particle trajectory, until box b_{i+1} either reaches the end of the kymograph or if two points in the box are of equal t but unequal x, or vice versa. If the latter occurs, the single particle trajectory is saved as contained in the aforementioned sequence of boxes, and the algorithm repeats by

forming a new sequence either from box b_{i+2} or from the start of a new trajectory, if the end of the kymograph (lengthwise) has been reached. Hence, regions in which multiple trajectories cannot be separated by more than d_t pixels are discarded. Since the optical contrast of EV trajectories is orders of magnitude higher than that of lipoproteins, the scattered light from lipoprotein trajectories in the near vicinity of EVs becomes vanishingly small after the pre-processing steps described in *Data pre-processing from raw data to kymograph,* and hence each EV trajectory corresponds to a single EV.

Additionally, the U-net and FCNN models are trained equivalently to the procedure described in *Image segmentation with U-net* and *Property calculation with FCNN,* with the following exceptions. First, to account for the induced flow in the channels, the models are trained on simulated particle trajectories within a large range of flow velocities and hence learn to ignore the effect of general flow on diffusion calculation. We note here that the accuracy of the diffusion calculation is dependent on flow velocity, as higher flow velocities in general lead to lower total measurement time of each separate biomolecule. This effect is however minimal in the low flow rates ($\sim 10 \mu m/s$) used for cell conditioned media analysis. Second, the range of *iOC* values the models are trained on is considerably larger: $iOC \approx 10^{-4}$ to 10^{-1} µm, and D values slightly smaller - $D \approx 0.1$ to 30 µm^2/s. Additionally, to improve accuracy below $iOC = 2.5 \cdot 10^{-3}$µm, we use the standard FCNN model described in *Property calculation with FCNN* in this regime. Third, since the channel size is larger and the trajectories of shorter length (in time), the models are trained on simulated kymographs of size 512x512 rather than 128x2048.

8. Evaluation of the data processing

To evaluate the performance of the data processing described in the SI sections "Removal of the background" and "Particle tracking", we have carried out benchmarking tests on ground truth data that were composed of experimentally recorded background signal, i.e., a time sequence of an intensity profile of a nanochannel filled with water (no biomolecules inside, I^0) combined with a generated response of biomolecules (I^r) with defined properties (iOC_{def} and D_{def}) that was simulated by the following procedure. At time zero, the positions of 10 biomolecules were randomly selected from the range of positions from -100 μm to +100 μm and their Brownian motion was recorded in time. At each time step (Δt), the position of each biomolecule was calculated from the previous position, as $x_i^p = x_{i-1}^p + R\sqrt{2D_{def}\Delta t}$, where R is a normally distributed random number with a mean of zero and a standard deviation of one. When a position of a biomolecule reached a value that was either lower than -100 μm or higher than +100 μm, it was reflected back in a specular fashion. The optical response of a biomolecule was then simulated as a gauss function, whose central position, width, and magnitude were defined by x_i^p, width of the diffraction limited spot (w_{DLS}), and iOC_{def}, respectively. To match the frame rate of the recorded data (200 frames per second) and to mimic the continuous illumination, every time frame of the simulated response with temporal length of $5\cdot10^{-3}$ s was averaged over 100 time-steps ($\Delta t = 5\cdot10^{-5}$ s) of the generated biomolecule positions, as

$$I_i^r(x) = \frac{1}{100}\sum_{j=1}^{100}\frac{iOC}{\sqrt{\pi}w_{DLS}}\sum_{p=1}^{20}\exp\left[-\left(\frac{x - x_{100(i-1)+j}^p}{w_{DLS}}\right)^2\right]$$

Equation 47

where x is a space coordinate that corresponds to the space coordinate of the recorded data. For each combination of values of $iOC_{def} = 10^{-4}, 2\cdot10^{-4}, 5\cdot10^{-4}, 10^{-3}, 2\cdot10^{-3}$ μm, and $D_{def} = 10$, 20, 50 μm^2/s, 10 different responses with a temporal length of 10000 frames were generated (selected examples are shown in **Fig. S10**). We note that the movement of a biomolecule within one time frame results in motion blur – broadening and shallowing of the intensity dip (**insets Fig. S10**).

The generated response was then combined with recorded background signal (**Fig. S11**) as $I = I^0 \cdot I^r$ and kymographs were created according to the procedure described in SI section "Removal of the background" (**Fig. S12**), positions of biomolecules were found using the algorithm described in SI section "Particle tracking algorithm". In the first step of the algorithm, the positions of all minima in the kymograph were found and their zero order intensity moments (m) were calculated. Since the m-values are calculated as a sum of intensities of a fixed fraction of a peak, and since the shape of the peak varies due to motion blur, m-values are also slightly dependent on the diffusivity (**Fig. S13**). Specifically, the mean value of m corresponding to D = 50 μm^2/s is 15% lower compared to the mean value of D = 10 μm^2/s. Nevertheless, motion blur has a minimal effect on determination of the *iOC* value, as it is calculated as integral value of the whole intensity dip that always remains constant.

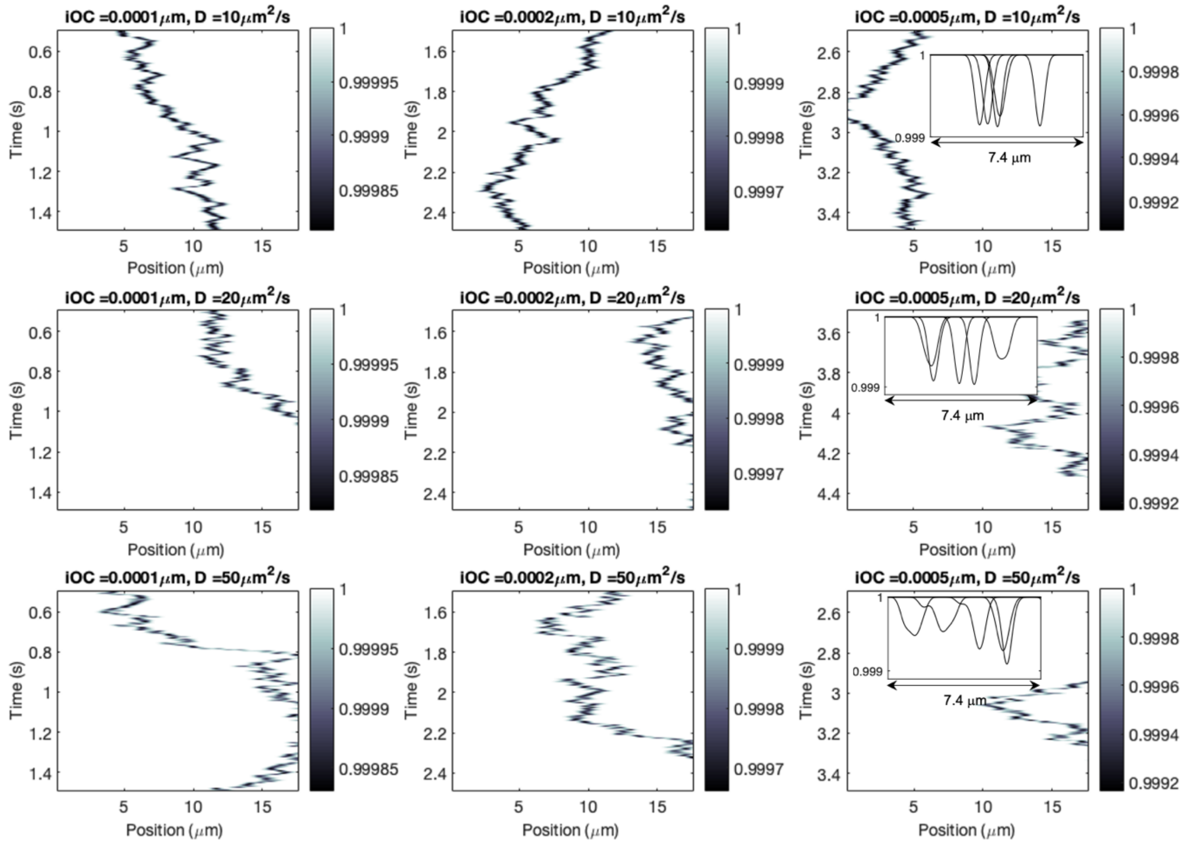

Fig. S10. Examples of the simulated response of biomolecules *defined by different combinations of iOC_{dif} and D_{dif}. The simulated data contained 844, 1036, and 1745 trajectories for data set corresponding to D_{dif} = 10, 20, 50 μm²/s with various temporal lengths (N frames).* ***Insets:*** *intensity profiles at 5 selected points in time illustrating motion blur - the broadening and shallowing of the intensity dip for molecules with high D.*

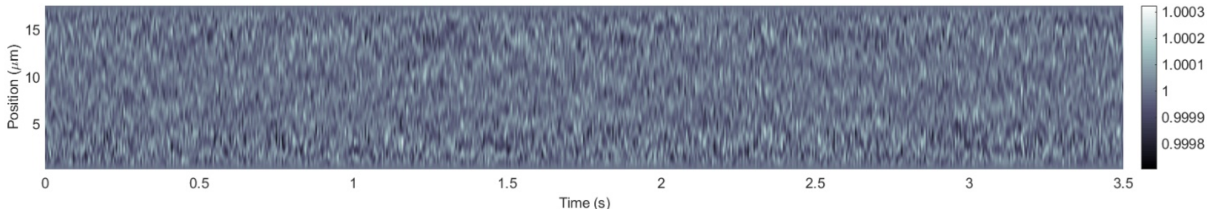

Fig. S11. *Example of kymograph corresponding to the experimentally recorded background signal from a nanochannel filled with pure PBS buffer, without the target molecules. When analysing this kymograph, correctly neither the standard algorithm nor the machine-learning algorithm identifies a molecule trajectory.*

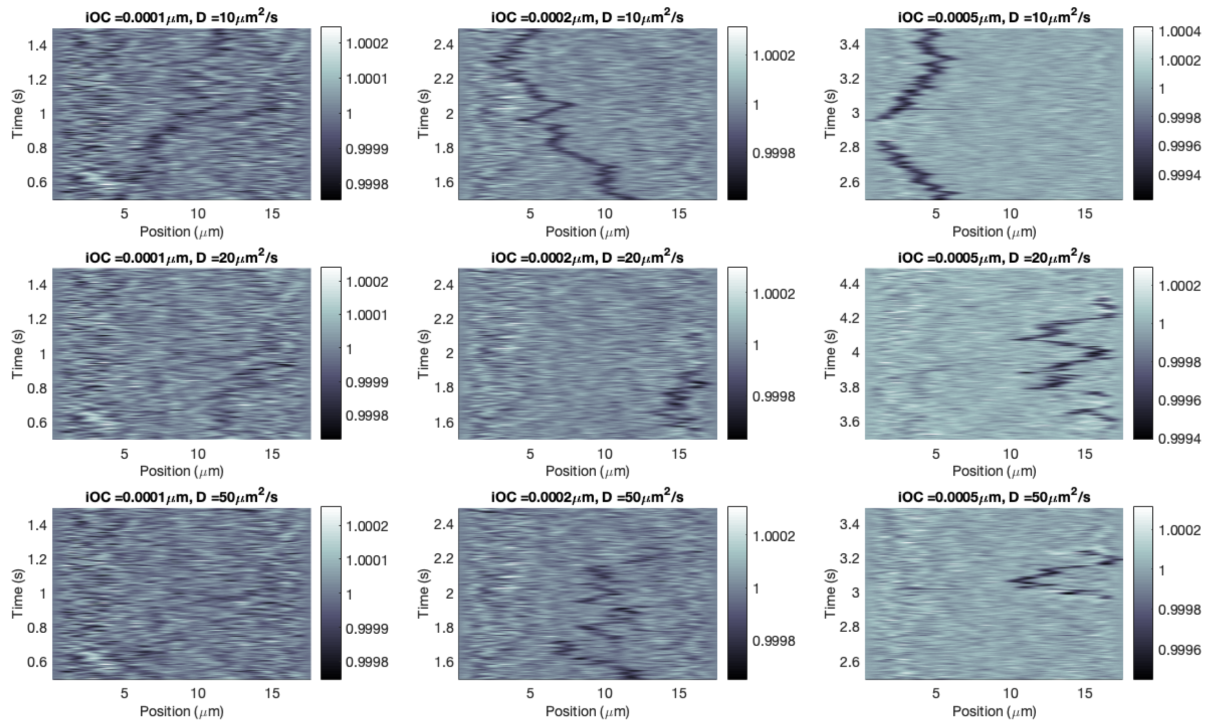

Fig. S12. Examples of kymographs corresponding to the simulated response of biomolecules *defined by different combinations of iOC_{def} and D_{def}.*

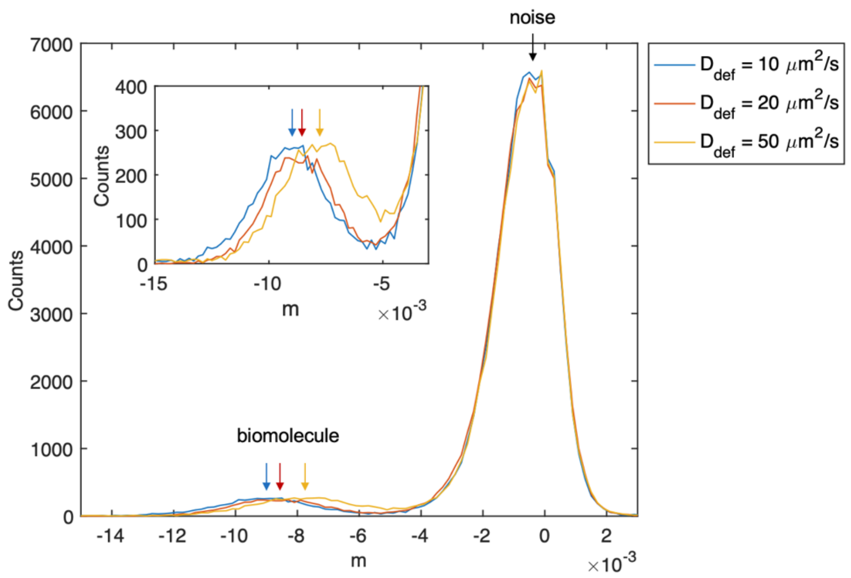

Fig. S13. Examples of zero order intensity moment histograms *obtained from a selected kymograph of 10000 frame temporal length corresponding to the simulated response of biomolecules defined by $iOC_{def} = 5 \cdot 10^{-4}$ µm and different D_{def} (parts of the selected kymograph are shown on Fig. S12).*

The determined values of *iOC* and *D* of all detected trajectories are shown as a scatter plot in **Fig. S14A**. The mean values of *iOC* and *D* (**Fig. S14B**) correspond to the position of the found peaks of the corresponding histograms of *iOC* (**Fig. S14C**) and *D* (**Fig. S14D**), where each trajectory is represented by *N* values of *iOC* and *D*, respectively. Clearly, the values identified by our procedure correspond to the ground truth very well, thereby corroborating the used data analysis protocol as a whole.

We also highlight that none of these tests revealed any false signals — i.e., no trajectories other than those simulated were found by the particle tracking algorithm. In addition, analysis of experimentally recorded background signal from a channel filled with pure PBS buffer, without the target molecules, did not reveal any false signal either (**Fig. S11**). That is the consequence of the fact that the used particle tracking algorithm discards all trajectories that have lower signal than 4 STD of the noise. In the case of machine learning, the models are trained on a wide variation of simulated noise distributions and train-validated against experimental channel noise to ensure that they do not find false signals in the experimentally relevant conditions that they have been validated against. Furthermore, we highlight that if the experimental conditions and noise distribution were to substantially change, the model could quickly be retrained by transfer-learning using the first few seconds of measurement of an empty channel and thereby always prevent false signal detection.

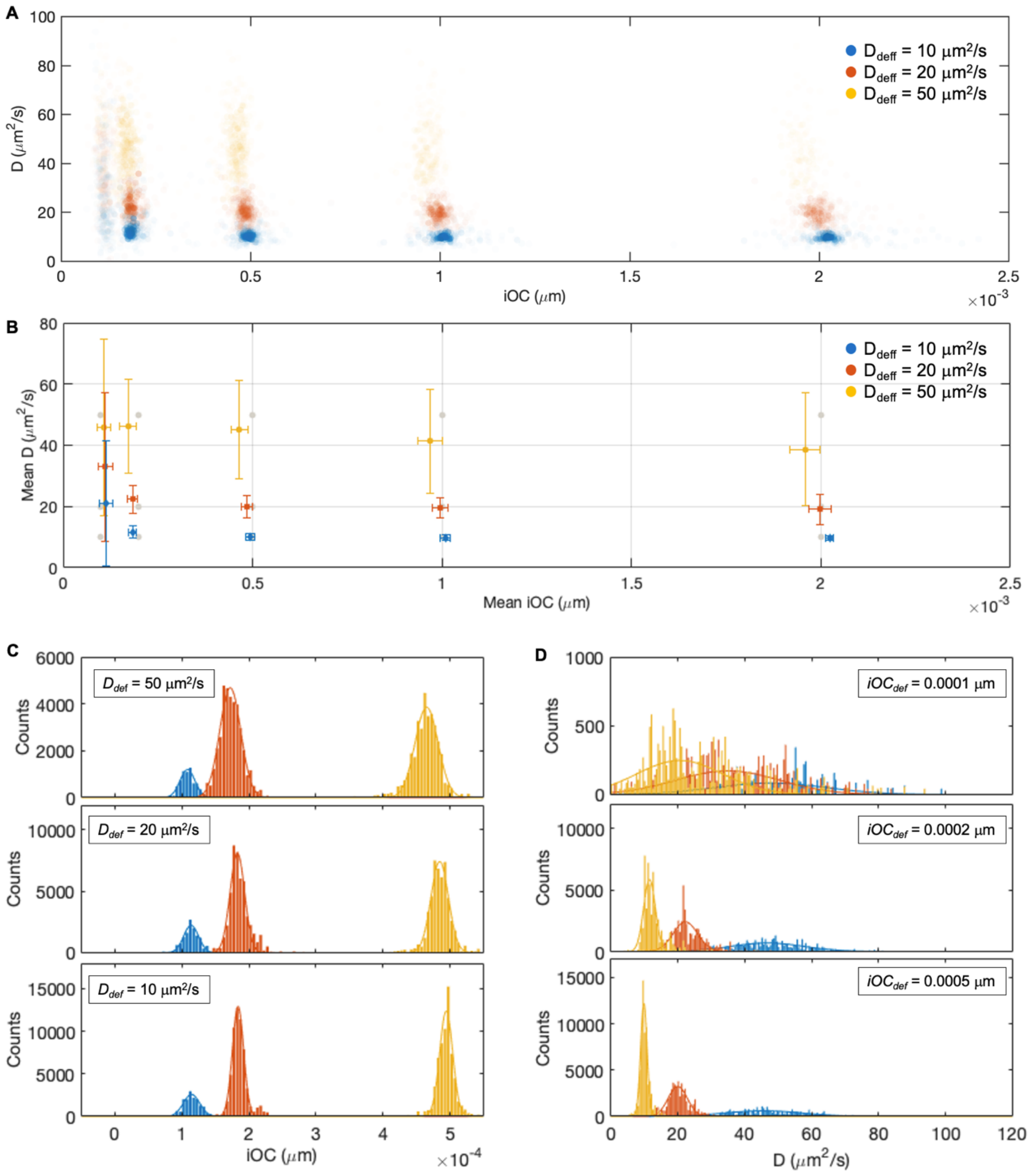

Fig. S14. Analysis of the artificially generated data set *comprised of experimentally recorded background signal and simulated response of biomolecules defined by a combination of values for* $iOC_{def} = 10^{-4}, 2{\cdot}10^{-4}, 5{\cdot}10^{-4}, 10^{-3}, 2{\cdot}10^{-3}$ *μm, and* $D_{def} = 10, 20, 50$ *μm²/s.* ***(A)*** *Scatter plot of iOC and D. Each dot corresponds to the values extracted from a single trajectory. The intensity of the colour indicates the number of frames the corresponding trajectory is comprised of. The highest intensity corresponds to N = 3300 frames.* ***(B)*** *Mean value of iOC and D. Error bars correspond to the resolution in iOC and D defined as FWHM of the peaks of the histograms. The presented values were derived from n = 844, 1036, and 1745 trajectories for data set corresponding to* $D_{dif} = 10, 20,$ *and 50 μm²/s, respectively. Examples of the* ***(C)*** *histograms of iOC and* ***(D)*** *histograms of D, corresponding to different combinations of* iOC_{def} *and* D_{def}*.*

9. Noise analysis

To evaluate the level of the noise in the system, we measured the standard deviation of the intensity in a kymograph corresponding to the background recorded from a nanochannel filled with pure PBS buffer (**Fig. S11**) over 500 s for a range of time frame averaging values (temporal length of one video frame). The camera was operated at 5400 frames per second. The experimental values are compared with the expected level of shot noise calculated as $1/\sqrt{N_t N_s N_{e^-}}$, where N_{e^-} is the well depth of photoelectrons in a single pixel ($N_{e^-} = 33\ ke^-$ for the Andor Zyla CMOS camera), N_s corresponds to spatial averaging (the signal was averaged over a diffraction-limited spot, $N_s = 20 \times 20$ pixels) and N_t corresponds to the time frame averaging. **Fig. S15** shows that the experimental values are very close to the theoretical shot-noise limit, which confirms that the measurement is shot-noise limited.

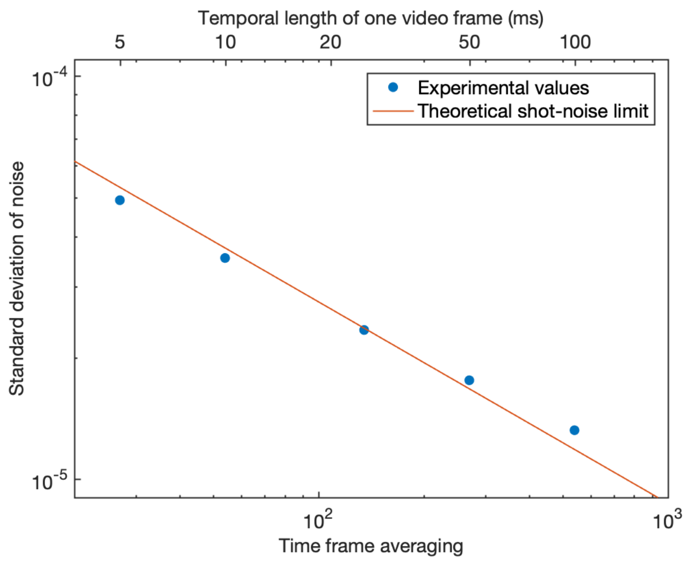

Fig. S15. Noise analysis. *Comparison of the experimentally measured standard deviation of the noise with the theoretical limit of the shot noise for a range of time averaging (temporal length of the video frame). The experimental values are very close to the theoretical limit, which confirms that the measurement is shot-noise limited.*

10. Resolution in molecular weight and hydrodynamic radius

The following section describes specific parameters and their interplay that influence the resolution of NSM in MW and R_S. The values of MW and R_S are inferred from the measured iOC and *D*, respectively. Therefore, we first discuss the factors that influence the resolution in iOC and *D*.

The uncertainty in iOC determination for each trajectory (standard deviation of the estimate for iOC, σ_{iOC}) is expected to follow

$$\sigma_{iOC} = \sigma_{iOC}^{0} \sqrt{\frac{1}{N}},$$

Equation 48

where σ_{iOC}^{0} is uncertainty in iOC determination from a single frame and *N* is the number of frames a single trajectory is composed of. The uncertainty in *D* determination for each trajectory (standard deviation of the estimate for *D*, σ_D) can be written (to first order in $1/N$) as[9]

$$\sigma_D \approx \sigma_D^{0} \sqrt{\frac{1}{N}},$$
$$\sigma_D^{0} = D\sqrt{6 + 4\varepsilon + 2\varepsilon^2}$$

Equation 49

where $\varepsilon = \delta^2/(D\Delta t) - 2R$, δ is the localization error, and $R = 1/6$ is a contribution corresponding to a motion blur for continuous illumination.

The resolution in *iOC* and *D* can be defined as the full-width-at-half-maximum of the peak in the histogram, where each detected trajectory from a data set corresponding to one type of a molecule is represented by *N* values of *iOC* and *D*, respectively. Since the number of frames varies stochastically, the width of the derived peak (and thus the resolution) depends on the distribution of the *N* values within one data set that can be described in terms of a probabilistic density function (f_N). f_N depends on the diffusivity, temporal length of a time frame, and the length of the field of view, and can be characterized by the mean value of *N*, $\bar{N} \approx (D\Delta t)^{-1/2}$, where Δt is the time step between the subsequent frames. Assuming that the measured values of both *iOC* and *D* are normally distributed numbers with standard deviations equal to σ_{iOC} and σ_D (Equation 48 and Equation 49), and that the number of the frames can be expressed in terms of the $f_N(N)$, the expected histogram of *iOC* and *D* can be written in a general form as

$$h(x) = \int \frac{f_N\sqrt{f_N}}{\sigma_x^{0}\sqrt{2\pi}} \exp\left(-\frac{1}{2} f_N \left(\frac{x - \bar{x}}{\sigma_x^{0}}\right)^2\right) dN$$

Equation 50

where *x* stands for either *iOC* or *D*. At first, to predict the shape of $h(x)$ that dictates the resulting resolution, we have simulated f_N for scenarios that correspond to experimental

conditions using the following procedure. At time frame $i = 1$, we have assumed a biomolecule at the position $x_1 = 0$. After that, the positios of the biomolecule in the subsequent time frame was calculated from the previous position, as $x_i = x_{i-1} + R\sqrt{2D\Delta t}$, where R is a normally distributed random number with a mean of zero and a standard deviation of one, and where $\Delta t = 5$ ms, and D =5 - 60 μm^2/s is the diffusivity of the biomolecule. If $x_i < 0$ or $x_i > L_{fow} =$ 17.5 μm, the biomolecule was considered to be out of the field of the view and the temporal length of a trajectory, $N = i$, was recorded. The process was repeated for 10^4 molecules. f_N was then plotted as the histogram of recorded N (an example for D = 20 μm^2/s is shown on **Fig. S16A**) and the mean value of N ($\overline{N}$) was calculated. Here we also take into account that all trajectories that have $N < 40$ are discarded by the particle tracking algorithm and are considered as noise. The obtained dependency of $\overline{N}$ on D (**Fig. S16B**) was then fitted to the function

$$\overline{N} = \frac{a}{D^b}, a = 1540, b = 0.57.$$

Equation 51

Subsequently, the expected histogram $h(x)$ for a general value $(x - \bar{x})/\sigma_x^0$ was calculated from the simulated f_N using Equation 50 (**Fig. S16C**) and the FWHM of the peak (corresponding to w_x/σ_x) was determined. Alternatively, the FWHM can be estimated from the gaussian fit, which can be highly relevant especially when evaluating statistics from a low number of trajectories. Interestingly, even though the shape of the peak is not strictly gaussian, the derived values of FWHM from the gaussian fit are very close to the real value (**Fig. S16D**). Therefore, we argue here that the gaussian fit can be used to estimate the resolution from the experimental data. The obtained dependency of w_x/σ_x on $\overline{N}$ (**Fig. S16D**) was then fitted to the function

$$w_x \approx \sigma_x^0 \frac{a}{\overline{N} + b}, a = 33, b = 62$$

Equation 52

which presents a phenomenological model of the resolution in both *iOC* and *D*. The resolution in *iOC* and *D* can then be written in terms of the mean number of frames, as

$$w_{iOC} \approx \sigma_{iOC}^0 \frac{a}{\overline{N} + b}, a = 33, b = 62$$

Equation 53

and

$$w_D \approx \sigma_D^0 \frac{a}{\overline{N} + b}, a = 33, b = 62,$$

Equation 54

respectively.

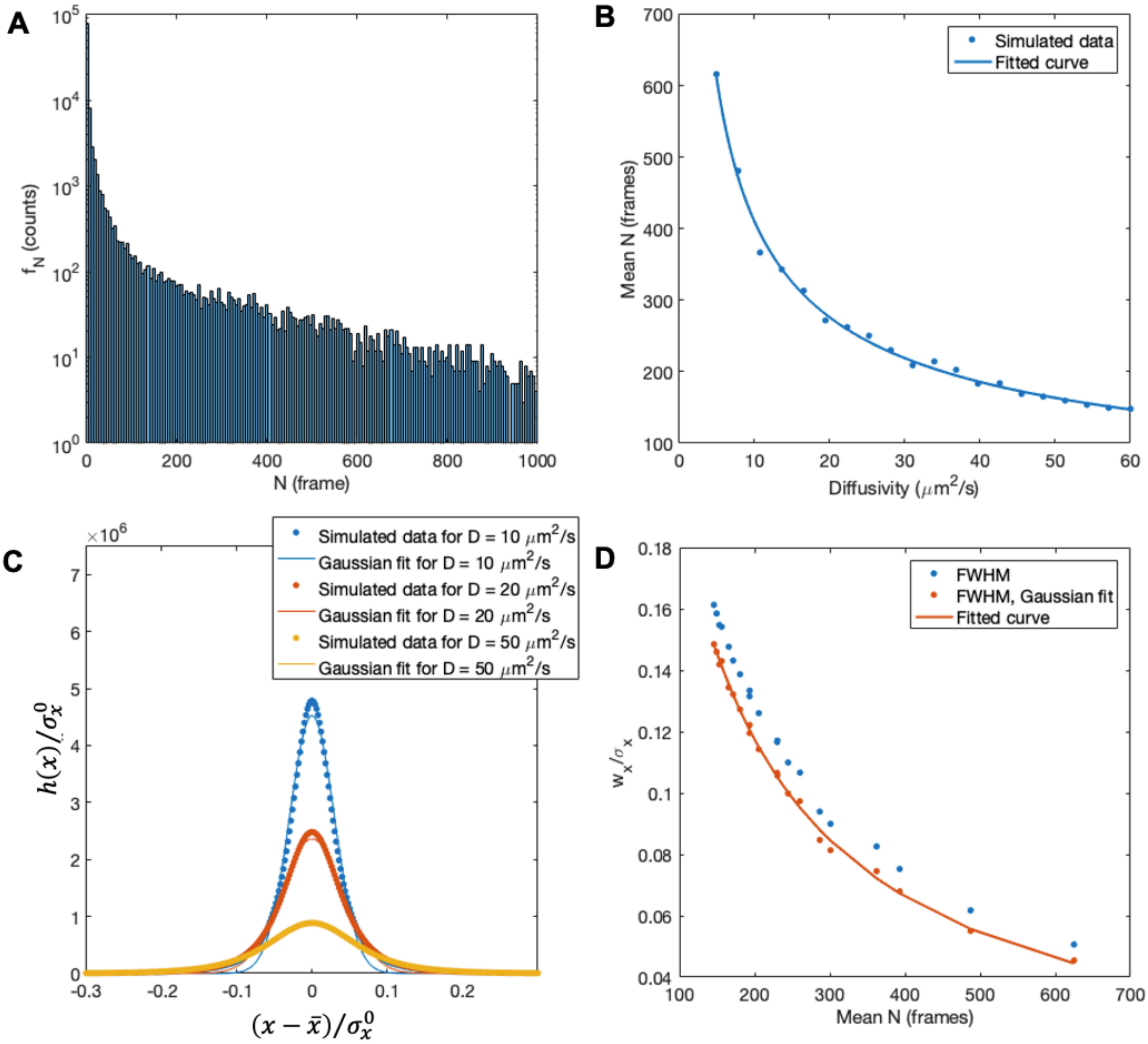

Fig. S16. Phenomenological model of the resolution. (A) *Probability density function, f_N, of temporal lengths of the trajectories (number of the frames, N) limited by the field of view L_{fow} = 17.5 μm that is recorded with the time step Δt = 5 ms of a biomolecule with D = 20 μm²/s.* ***(B)*** *Dependency of the mean value of N on diffusivity of a biomolecule.* ***(C)*** *Expected histogram of a normalized value $(x-\bar{x})/\sigma_x^0$ for biomolecules with diffusivity D = 10, 20, and 50 μm²/s.* ***(D)*** *Dependency of the mean value of N on normalized FWHM of the expected histogram, w_x/σ_x^0.*

Having established the theoretical background, we now discuss the specific resolution in *iOC* and *D* obtained from three data sets analyzed by the standard analysis (SA), described in Methods: "Removal of the background" and "Particle tracking algorithm": simulated data corresponding to biomolecules defined by the combinations of values iOC_{def} and D_{def} (**Fig. S12**, more details in section "Evaluation of the data processing") and the experimental data obtained for different proteins and DNAs in Channel I and II (**Fig. 2** in the main text). It can be seen that the resolution in both *iOC* and *D* exhibits a complex behavior. The resolution in *iOC* (**Fig. S17A**) increases both with diffusivity and *iOC* of a pertinent biomolecule, whereas the resolution in *D* (**Fig. S17B**) increases with diffusivity and decreases with increasing *iOC* of a pertinent biomolecule. To explain this behavior, we further discuss the main factors that influence both resolutions.

The resolution in *iOC* is directly proportional to the factor σ^0_{iOC} that relates to the noise in the kymograph and that can be calculated from w_{iOC} (**Fig. S17A**) and $\bar{N}$ (**Fig. S17E**), using the phenomenological model (Equation 53). The values determined for each data set (**Fig. S17C**) are compared with the value corresponding to the ideal system that is limited by the shot noise and that can be estimated as $\sigma^0_{iOC} = w/\sqrt{N_e\text{-}N_t N_s}$, where w = 1.18 μm (corresponding to 40 pixels) is the length of the nanochannel from which the *iOC* is calculated, $N_t = 25$ frames and $N_s = 40 \times 20$ pixels corresponds to temporal and spatial averaging, and $N_{e^-} = 33\ ke^-$ is the well depth of a single pixel. It can be seen that σ^0_{iOC} corresponding to the biomolecules from the data sets increases with *iOC* and the theoretical limit corresponding to the shot noise was reached only in the case of a biomolecule with the lowest *iOC* (200 bp DNA). That can be attributed to the additional noise introduced by the background removal algorithm.

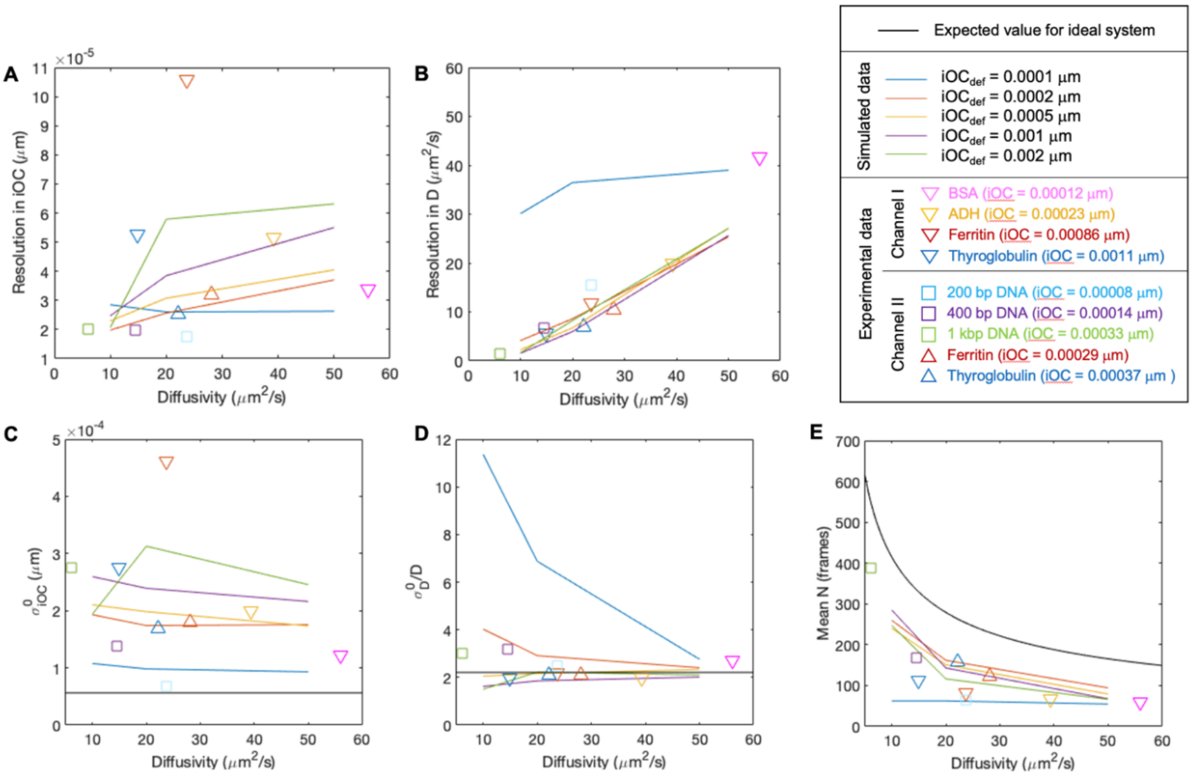

Fig. S17. Analysis of the resolution in iOC and D *for a series of data sets corresponding to: simulated data corresponding to biomolecules defined by the combinations of values iOC$_{def}$ and D$_{def}$ and the experimental data obtained for different proteins and DNAs in Channel I and II and analysed by SA.* ***(A)*** *Resolution in iOC (w_{iOC}).* ***(B)*** *Resolution in D (w_D).* ***(C)*** *A factor σ^0_{iOC} that relates to the noise in the kymograph and that was calculated from w_{iOC} and $\bar{N}$ using Equation 53. The black line corresponds to the values expected for the ideal system limited by shot noise.* ***(D)*** *A factor σ^0_D/D that relates to the localization error of the particle tracking algorithm and that was calculated from w_D and $\bar{N}$ using Equation 54. The black line corresponds to the ideal system without the localization error.* ***(E)*** *The mean number of the frames from which the trajectories in a data set were composed of ($\bar{N}$). The black line corresponds to the expected values (Equation 51).*

The resolution in D is directly proportional to the factor σ_D^0 that linearly increases with diffusivity and that is also related to the localization precision of the particle tracking algorithm (Equation 49), and that can be calculated from w_D (**Fig. S17B**) and $\bar{N}$ (**Fig. S17E**) using the phenomenological model (Equation 54). The values determined for each data set (**Fig. S17D**) are compared with the values corresponding to the ideal system with no localization error ($\delta = 0$). It can be seen that for high iOC the determined values for the data sets are very close to the case of the ideal system that indicates that for this regime, the localization error is negligible. However, with decreasing iOC and decreasing D, the values of σ_D^0 can be up to about 6 times higher compared to the ideal system which suggests that the localization error reduces the resolution in D in this regime. However, this behavior is expected for signals approaching the standard deviation of the noise in the system.

Moreover, the resolution in both iOC and D are influenced by the distribution of the temporal lengths of the trajectories a pertinent data set is composed of and that can be characterized by $\bar{N}$. The values of $\bar{N}$ derived for each data set (**Fig. S17E**) are compared with the expected values calculated using the phenomenological model (Equation 51). It can be seen that the derived values are lower than expected and show additional dependency on iOC that is not expected. This can be attributed to the fact that the trajectories with higher iOC suffer from higher variation in iOC than expected (see **Fig. S17D**) and therefore failing to fulfil the condition set on variation of iOC expected from an identical molecule. As a result, a single trajectory is separated into multiple shorter ones.

In addition, it has to be noted that the behavior for both simulated and experimentally obtained data sets are quantitively and qualitatively comparable, which confirms that the experimental data sets fulfil all the conditions that were applied to the simulated data set (e.g., free movement of the molecules). The exception is the ferritin and ADH systems, whose w_{iOC} (**Fig. S17A**) is about two times higher than for simulated molecules with comparable properties. This can, however, be attributed to the presence of additional populations containing different amounts of iron atoms in the case of ferritin (more details in the main text) and a possible population of dimers in the case of ADH.

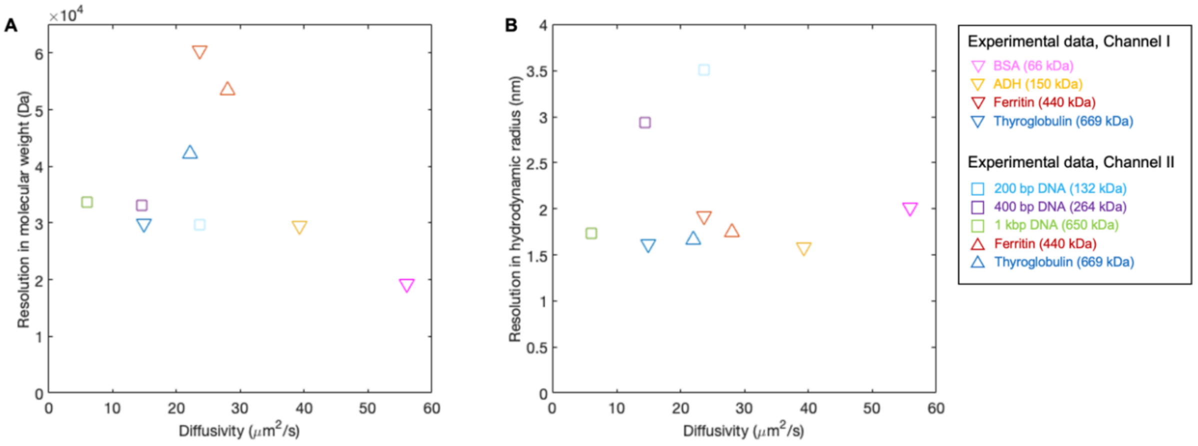

Fig. S18. *(**A**) Resolution in molecular weight and (**B**) resolution in hydrodynamic radius for experimental data obtained for different proteins and DNAs in Channel I and II and analysed by SA.*

The determined value of iOC can be translated into molecular weight using Equation 1 in the main text. The resolution in MW, w_{MW}, is then linearly dependent on the resolution in iOC and linearly increases with the cross-sectional area of the nanochannel, $w_{MW} = w_{iOC} \cdot A/(\bar{n}\alpha_{MW})$. We note here that these predictions assume that the shot noise level remains the same, i.e., the intensity and temporal averaging remain the same. In other words, downsizing of the nanochannel cross section improves the optical performance, but only to the level where the intensity of the scattered light saturates the camera at its maximal frame rate. In addition, downsizing the nanochannel cross section presents a challenge for nanofabrication, sets limits on the maximal size of molecules that can enter the channel and be analyzed, and increases the risk of clogging. However, in the section "Surface passivation by supported lipid bilayer" we show that the risk for clogging can be minimized by surface modification of the nanochannel walls by, e.g., a lipid bilayer, to avoid adsorption of molecules on the surface. In addition, in the section "Analysis of extracellular vesicles in conditioned cell culture medium" we show that the nanochannel dimensions can be tailored to accommodate even BNPs, such as EVs, without any obvious problems related to clogging. Looking forward, an analysis covering a wide range of molecular weights could be enabled by a series of nanochannels with varying sizes used in parallel. To prevent the clogging of the smaller nanochannels by larger molecules present in the sample, we propose that on-chip sorting systems[21] could be utilized.

The determined value of D can be translated into hydrodynamic radius using Equation 2 in the main text. The resolution in Rs, w_{R_S}, is then linearly dependent on the resolution in D and is indirectly proportional to the diffusivity of the biomolecule, $w_{R_S} \sim w_D \cdot D^{-1}$. Since the $w_D \sim D$ (Equation 49 and Equation 54) for systems with negligible localization error, the resolution in hydrodynamic radius for most of the studied molecules is around 2 nm (**Fig. S18B**). For the two smallest DNAs (200 kDa and 400 kDa) the resolution is worse (about 3 and 3.5 nm), due to the higher localization error specific for the regime of lower *iOC* and *D*.

11. The ferritin system

It is reported in the literature that the *MW* of ferritin may vary between 450 and 702 kDa, depending mostly on the amount of stored iron[22]. At the same time, exact and unbiased characterization of the *MW* of ferritin is challenging with existing methods, since its outer dimensions are independent of iron content[22]. Analysis is complicated further by the presence of reported up to five types of dimers with different interaction strengths[23]. This is unfortunate because accurate determination of iron content in ferritin from patients has recently been suggested as biomarker for clinical analysis of iron deficiency/overload related to infection, inflammation, and cancer[24].

We investigate the ferritin system in more detail in **Fig. S19**, and include data from experiments in two additional nanochannels (Channels III and IV, **Extended Data Fig. 3C, D**) with different cross-section areas ($A_{III} = 100 \times 15$ nm^2, and $A_{IV} = 145 \times 27$ nm^2) analyzed by SA. The two-dimensional histogram of inferred *MW* and R_S (**Fig. S19A)** and corresponding one-dimensional histogram of *MW* (**Fig. S19B)** reveal three different populations, marked as $M_{1\text{-}3}$, with *MW* ranging between ~ 500 and 900 kDa. Notably, they all have similar R_S ~ 6 nm, agreeing well with reported values for ferritin monomers[25,26]. In addition, a small number of molecules with *MW* > 900 kDa can be seen (marked "Dim" in **Fig. S19A**). They exhibit a mean R_S ~ 8 nm, agreeing well with the value reported for ferritin dimers[26]. This observation is further corroborated by plotting R_S histograms for four different *MW* ranges that reveal a single R_S population for the three *MW* populations "$M_{1\text{-}3}$", with very good agreement between the different nanochannels (**Fig. S19C-E)**, and additional R_S populations only for the data set obtained with Channel II (**Fig. S19F**), which had the largest cross-section. We thus conclude that the "$M_{1\text{-}3}$" populations correspond to monomers with different amounts of iron, since this alters their polarizability[27] and thus *iOC* and inferred *MW,* and that the "Dim" population corresponds to dimers. The reason why the "Dim" population is only present in the largest Channel II is likely that dimers are effectively filtered by Channels I, III and IV, due to their smaller cross-sections.

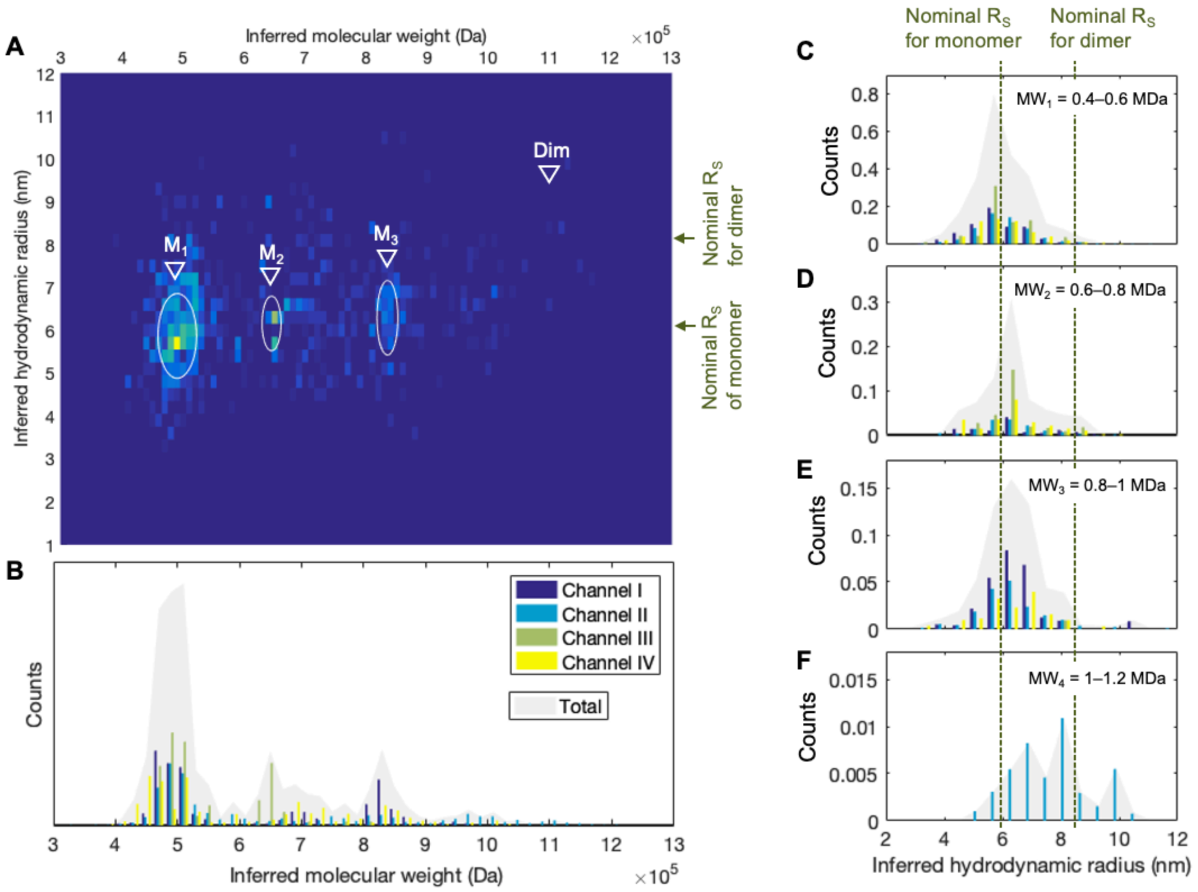

Fig. S19. Detailed analysis of the ferritin system. (A) *Two-dimensional histograms of inferred MW and R_S values of ferritin molecules detected in Channels I-IV using SA.* ***(B)*** *One-dimensional histogram of MW from the data shown in (A).* ***(C-F)*** *Histograms of R_S from data shown in (A) equidistantly separated into four regions with different ranges of MW,* ***(C)*** *MW_1 = 0.4 – 0.6 MDa,* ***(D)*** *MW_2 = 0.6 – 0.8 MDa,* ***(E)*** *MW_3 = 0.8 – 1 MDa,* ***(F)*** *MW_4 = 1 – 1.2 MDa.*

12. Characterization of the conditioned cell culture medium using Nanoparticle Tracking Analysis

In order to validate the results of the NSM analysis of the conditioned cell culture medium, we have carried out a particle size distribution measurement of the same sample using Nanoparticle Tracking Analysis (NTA, **Fig. S20**), with which we identified a population of extracellular vesicles (EVs) whose mode value of $R_S = 37.9$ nm is in very good agreement with the mode value identified by NSM ($R_S = 34$ nm, **Fig. 5D**). The absence of the population of smaller particles (lipoproteins) in the NTA data is the consequence of these small particles being below the limit of detection of the method.

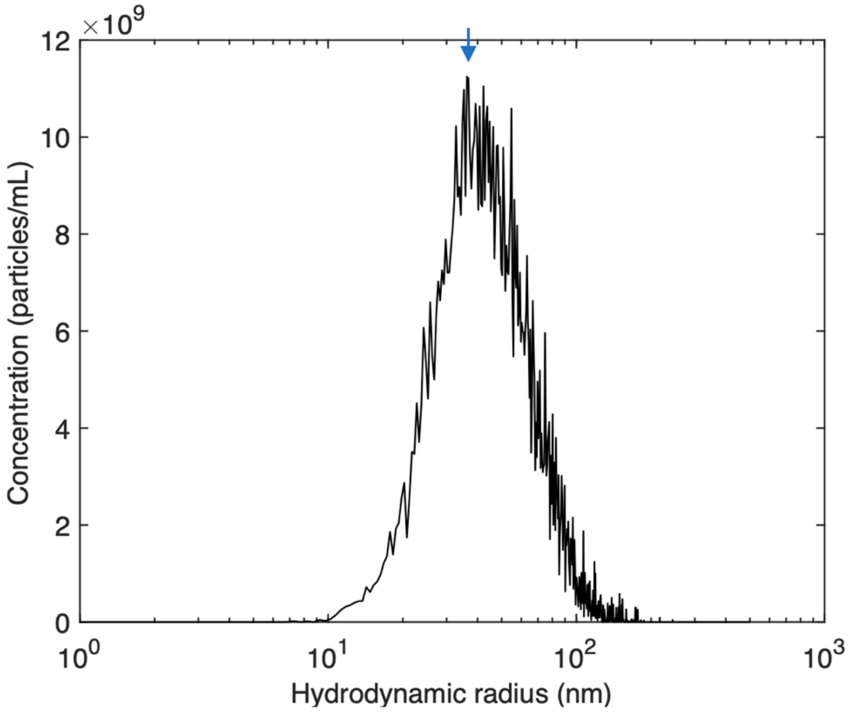

Fig. S20. Size distribution of BNPs *present in the conditioned cell culture medium characterized by NTA. The population of EVs is marked by the blue arrow.*

References

1 Bohren, C. F. & Huffman, D. R. *Absorption and Scattering of Light by Small Particles*. (Wiley-VCH, 1983).

2 Mangini, F. & Tedeschi, N. Scattering of an electromagnetic plane wave by a sphere embedded in a cylinder. *J Opt Soc Am A* **34**, 760-769, doi:10.1364/Josaa.34.000760 (2017).

3 Markel, V. A. Introduction to the Maxwell Garnett approximation: tutorial. *J Opt Soc Am A* **33**, 1244-1256, doi:10.1364/Josaa.33.001244 (2016).

4 Persson, F. *et al.* Lipid-based passivation in nanofluidics. *Nano Lett* **12**, 2260-2265, doi:10.1021/nl204535h (2012).

5 Barer, R. & Joseph, S. Refractometry of Living Cells .1. Basic Principles. *Q J Microsc Sci* **95**, 399-423 (1954).

6 Dechadilok, P. & Deen, W. M. Hindrance factors for diffusion and convection in pores. *Ind Eng Chem Res* **45**, 6953-6959, doi:10.1021/ie051387n (2006).

7 Olsen, E. *et al.* Diffusion of Lipid Nanovesicles Bound to a Lipid Membrane Is Associated with the Partial-Slip Boundary Condition. *Nano Lett* **21**, 8503-8509, doi:10.1021/acs.nanolett.1c02092 (2021).

8 Sbalzarini, I. F. & Koumoutsakos, P. Feature point tracking and trajectory analysis for video imaging in cell biology. *J Struct Biol* **151**, 182-195, doi:10.1016/j.jsb.2005.06.002 (2005).

9 Vestergaard, C. L., Blainey, P. C. & Flyvbjerg, H. Optimal estimation of diffusion coefficients from single-particle trajectories. *Phys Rev E* **89**, doi:ARTN 022726
10.1103/PhysRevE.89.022726 (2014).

10 Midtvedt, B. *et al.* Quantitative digital microscopy with deep learning. *Applied Physics Reviews* **8**, 011310 (2021).

11 Ronneberger, O., Fischer, P. & Brox, T. U-Net: Convolutional Networks for Biomedical Image Segmentation. *CoRR* **abs/1505.04597**, arXiv:1505.04597 (2015).

12 Redmon, J. & Farhadi, A. YOLOv3: An Incremental Improvement. *CoRR* **abs/1804.02767**, arXiv:1804.02767 (2018).

13 Goodfellow, I. J. *et al.* Generative Adversarial Networks. arXiv:1406.2661 (2014).

14 Kingma, D. P. & Ba, J. Adam: A Method for Stochastic Optimization. arXiv:1412.6980 (2017).

15 Netron: Visualizer for neural network, deep learning and machine learning models. *https://www.lutzroeder.com/ai*.

16 Rivenson, Y. *et al.* PhaseStain: the digital staining of label-free quantitative phase microscopy images using deep learning. *Light Sci Appl* **8**, 23, doi:10.1038/s41377-019-0129-y (2019).

17 Noren, E. L. Pytorch-yolov3. https://github.com/eriklindernoren/PyTorch-YOLOv3 (2019).

18 Redmon, J. Darknet: Open Source Neural Networks in C. http://pjreddie.com/darknet/ (2013–2016).

19 Ghosh, A., Sumpter, B., Dyck, O., Kalinin, S. & Ziatdinov, M. Ensemble learning-iterative training machine learning for uncertainty quantification and automated experiment in atom-resolved microscopy. *npj Computational Materials* **7**, doi:https://doi.org/10.1038/s41524-021-00569-7 (2021).

20 Lakshmanan, V., Görner, M. & Gillard, R. *Practical Machine Learning for Computer Vision*. 138 (O'Reilly Media, Inc., 2021).
21 Bayareh, M. An updated review on particle separation in passive microfluidic devices. *Chemical Engineering & Processing: Process Intensification* **153**, 107984 (2020).
22 Harrison, P. M. & Arosio, P. Ferritins: Molecular properties, iron storage function and cellular regulation. *Bba-Bioenergetics* **1275**, 161-203, doi:Doi 10.1016/0005-2728(96)00022-9 (1996).
23 Yang, D. W., Matsubara, K., Yamaki, M., Ebina, S. & Nagayama, K. Heterogeneities in Ferritin Dimers as Characterized by Gel-Filtration, Nuclear-Magnetic-Resonance, Electrophoresis, Transmission Electron-Microscopy, and Gene Engineering Techniques. *Bba-Protein Struct M* **1206**, 173-179, doi:Doi 10.1016/0167-4838(94)90205-4 (1994).
24 Namaste, S. M. L. *et al.* Adjusting ferritin concentrations for inflammation: Biomarkers Reflecting Inflammation and Nutritional Determinants of Anemia (BRINDA) project. *Am J Clin Nutr* **106**, 359s-371s, doi:10.3945/ajcn.116.141762 (2017).
25 La Verde, V., Dominici, P. & Astegno, A. Determination of Hydrodynamic Radius of Proteins by Size Exclusion Chromatography. *Bio-Protocol* **7**, doi:ARTN e2230
10.21769/BioProtoc.2230 (2017).
26 Petsev, D. N., Thomas, B. R., Yau, S. & Vekilov, P. G. Interactions and aggregation of apoferritin molecules in solution: effects of added electrolytes. *Biophys J* **78**, 2060-2069, doi:10.1016/s0006-3495(00)76753-1 (2000).
27 Gupta, R., Alamrani, N. A., Greenway, G. M., Pamme, N. & Goddard, N. J. Method for Determining Average Iron Content of Ferritin by Measuring its Optical Dispersion. *Anal Chem* **91**, 7366-7372, doi:10.1021/acs.analchem.9b01231 (2019).
