# Supplementary material for: Label-free nanofluidic scattering microscopy of size and mass of single diffusing molecules and nanoparticles
Source: Nat Methods. 2022 May 30;19(6):751–8. doi: 10.1038/s41592-022-01491-6 (PMC9184284; doi:10.1038/s41592-022-01491-6)
Supplement: Supplementary file 2 — Reporting Summary [file 41592_2022_1491_MOESM2_ESM.pdf]

nature research

Corresponding author(s): Barbora Spackova & Christoph Langhammer

Last updated by author(s): 31/3/22

Reporting Summary

Nature Research wishes to improve the reproducibility of the work that we publish. This form provides structure for consistency and transparency in reporting. For further information on Nature Research policies, see our Editorial Policies and the Editorial Policy Checklist.

Statistics

For all statistical analyses, confirm that the following items are present in the figure legend, table legend, main text, or Methods section.

n/a	Confirmed	
☐	☒	The exact sample size (n) for each experimental group/condition, given as a discrete number and unit of measurement
☐	☒	A statement on whether measurements were taken from distinct samples or whether the same sample was measured repeatedly
☒	☐	The statistical test(s) used AND whether they are one- or two-sided<br>*Only common tests should be described solely by name; describe more complex techniques in the Methods section.*
☒	☐	A description of all covariates tested
☒	☐	A description of any assumptions or corrections, such as tests of normality and adjustment for multiple comparisons
☐	☒	A full description of the statistical parameters including central tendency (e.g. means) or other basic estimates (e.g. regression coefficient) AND variation (e.g. standard deviation) or associated estimates of uncertainty (e.g. confidence intervals)
☒	☐	For null hypothesis testing, the test statistic (e.g. F, t, r) with confidence intervals, effect sizes, degrees of freedom and P value noted<br>*Give P values as exact values whenever suitable.*
☒	☐	For Bayesian analysis, information on the choice of priors and Markov chain Monte Carlo settings
☒	☐	For hierarchical and complex designs, identification of the appropriate level for tests and full reporting of outcomes
☒	☐	Estimates of effect sizes (e.g. Cohen's d, Pearson's r), indicating how they were calculated

Our web collection on statistics for biologists contains articles on many of the points above.

Software and code

Policy information about availability of computer code

Data collection	The data were collected using a custom code NSM-DeviseControl v1.0 that can be found at gitlab.com/langhammerlab/NSM-DeviceControl.
Data analysis	The data were analyzed by a custom code NSM-SA v 1.0 utilizing SA described in SI sections 5-6 and a custom code NSM-ML v1.0 utilizing ML described in SI section 7. The codes can be found at gitlab.com/langhammerlab/NSM-SA and gitlab.com/langhammerlab/NSM-ML.

For manuscripts utilizing custom algorithms or software that are central to the research but not yet described in published literature, software must be made available to editors and reviewers. We strongly encourage code deposition in a community repository (e.g. GitHub). See the Nature Research guidelines for submitting code & software for further information.

Data

Policy information about availability of data

All manuscripts must include a data availability statement. This statement should provide the following information, where applicable:

- Accession codes, unique identifiers, or web links for publicly available datasets
- A list of figures that have associated raw data
- A description of any restrictions on data availability

A sample data are packaged with the code and can be found at gitlab.com/langhammerlab/NSM-SA. Source data are provided with this paper.

Field-specific reporting

Please select the one below that is the best fit for your research. If you are not sure, read the appropriate sections before making your selection.

☒ Life sciences ☐ Behavioural & social sciences ☐ Ecological, evolutionary & environmental sciences

For a reference copy of the document with all sections, see nature.com/documents/nr-reporting-summary-flat.pdf

Life sciences study design

All studies must disclose on these points even when the disclosure is negative.

Sample size	This is not relevant to us because we draw no "life science" conclusions based on the results. We only report the information of the measured sample without any general conclusions about samples taken from the natural world.
Data exclusions	No data were excluded.
Replication	To verify the reproducibility, the data corresponding to the ADH molecule were collected using three different nanochannels with the same geometry. No statistically relevant differences between the results from different nanochannels were found.
Randomization	No randomization was done because we have not performed any "treatment" or modification of samples. The samples are taken from specific sources outlined in the main text.
Blinding	Same as randomization.

Reporting for specific materials, systems and methods

We require information from authors about some types of materials, experimental systems and methods used in many studies. Here, indicate whether each material, system or method listed is relevant to your study. If you are not sure if a list item applies to your research, read the appropriate section before selecting a response.

Materials & experimental systems

n/a	Involved in the study	
☒	☐	Antibodies
☐	☒	Eukaryotic cell lines
☒	☐	Palaeontology and archaeology
☒	☐	Animals and other organisms
☒	☐	Human research participants
☒	☐	Clinical data
☒	☐	Dual use research of concern

Methods

n/a	Involved in the study	
☒	☐	ChIP-seq
☒	☐	Flow cytometry
☒	☐	MRI-based neuroimaging

Eukaryotic cell lines

Policy information about cell lines

Cell line source(s)	SH-SY5Y human neuroblastoma acquired from ECACC.
Authentication	The SH-SY5Y cell line was authenticated by Eurofin Genomics by STR profiling according to ANSI/ATCC standard ASN-002.
Mycoplasma contamination	The SH-SY5Y cells were regularly tested for mycoplasma contamination by standardised qPCR test by Eurofins Genomics under ISO17025 accreditation. The test results were always negative showing that the cell cultures used are mycoplasma free.
Commonly misidentified lines (See ICLAC register)	None; our cell line is not in the ICLAC register.
